# Understanding the treatment response and resistance to targeted therapies in non-small cell lung cancer: clinical insights and perspectives

**DOI:** 10.3389/fonc.2024.1387345

**Published:** 2024-07-11

**Authors:** Hang Zhang, Yingying Zhang, Yingying Zhu, Tian Dong, Zheng Liu

**Affiliations:** ^1^ Department of Hematology, Institute of Hematology, West China Hospital of Sichuan University, Chengdu, China; ^2^ Department of Neurology, West China Hospital of Sichuan University, Chengdu, China; ^3^ Department of Respiratory and Critical Care Medicine, West China Hospital, Sichuan University, Chengdu, China; ^4^ Department of Thoracic Surgery, West China Hospital, Sichuan University, Chengdu, China

**Keywords:** non-small cell lung cancer, targeted therapy, treatment response, drug resistance, tyrosine kinase inhibitor

## Abstract

Lung cancer remains the leading cause of mortality worldwide. Non-small cell lung cancer (NSCLC) is the most common subtype of lung cancer with a generally poor prognosis. In recent years, advances in targeted therapy and sequencing technology have brought significant improvement in the therapeutic outcomes of patients with advanced NSCLC. Targeted inhibitors directed against specific mutated or rearranged oncogenes, such as epidermal growth factor receptor (*EGFR*), anaplastic lymphoma kinase (*ALK*), and receptor tyrosine kinase ROS proto-oncogene 1(*ROS1*) among others, exhibit promising anti-tumor activity. Unfortunately, some patients develop acquired resistance and disease progression soon after initial remission. Despite the continuous development of new drugs and strategies to overcome drug resistance, it is still a major challenge in the treatment of NSCLC. The landscape of targeted therapy for NSCLC is evolving rapidly in response to the pace of scientific research. This study aimed to provide a comprehensive review of tumor target antigens and agents related to targeted therapy in NSCLC.

## 1 Introduction

In recent years, targeted therapy has achieved significant success in advanced non-small cell lung cancer (NSCLC). Patients with metastatic lung cancer who qualify for targeted therapies now experience prolonged survival, with 5-year survival rates ranging from 15% to 60%, contingent on the specific biomarker identified (1–4). Consequently, molecular and immune biomarker testing of lung cancer specimens is crucial to identifying potentially effective targeted treatments, especially in patients with metastatic NSCLC (3–7). It aims to alleviate symptoms, decrease tumor burden, and improve overall survival (OS).

Classic actionable biomarkers included various genetic alterations that are the targets of several tyrosine kinase inhibitors (TKI) such as anaplastic lymphoma kinase (*ALK*) rearrangement, V-RAF mouse sarcoma virus oncogene homolog B1 (*BRAF*) p.V600E mutation, epidermal growth factor receptor (*EGFR*) mutation, erb-b2 receptor tyrosine kinase 2 (*ERBB2*, also known as human epidermal growth factor receptor 2, *HER2*) mutation, Kirsten rat sarcoma virus (*KRAS*) mutation, mesenchymal-epithelial transition factor (*MET*) exon 14 (METex14) skipping mutation, neurotrophic tyrosine receptor kinase 1/2/3 (*NTRK1/2/3*) gene fusion, rearranged in transfection (*RET*) rearrangement, receptor tyrosine kinase ROS proto-oncogene 1(*ROS1*) rearrangement, and high-level *MET* amplification. These gene alterations typically occur in a non-overlapping manner. However, 1%–3% of patients may have coexistence of more than one of these biomarkers (8).

Here we summarize the essential therapeutic targets and targeted drugs for NSCLC (**Table 1**) and provide insights into the treatment response and resistance mechanisms associated with targeted therapies.

**Table 1:** Summary of US FDA approved targeted therapies for non-small cell lung cancer.

Drug	Approved year	Target	Indication
Gefitinib	2003	EGFR	EGFR mutant NSCLC
Erlotinib	2004	EGFR	EGFR mutant NSCLC
Crizotinib	2011	ALK and ROS1	ALK and ROS1positive NSCLC
Afatinib	2013	EGFR, HER2 and HER4	EGFR mutant NSCLC
Trametinib	2013	MEK1/2	BRAF mutant NSCLC
Dabrafenib	2013	BRAF	BRAF mutant NSCLC
Ceritinib	2014	ALK, IGF-1R and ROS1	ALK positive NSCLC
Osimertinib	2015	EGFR	EGFR mutant NSCLC
Alectinib	2015	ALK and RET	ALK positive NSCLC
Brigatinib	2017	ALK, ROS1, IGF-1R and EGFR	ALK positive NSCLC
Dacomitinib	2018	EGFR, HER2 and HER4	EGFR mutant NSCLC
Lorlatinib	2018	ALK and ROS1	ALK positive NSCLC
Entrectinib	2019	TRKA/B/C, ROS1, ALK	NTRK positive NSCLC
Capmatinib	2020	MET	MET mutant NSCLC
Selpercatinib	2020	RET	RET positive NSCLC
Pralsetinib	2020	RET	RET positive NSCLC
Tepotinib	2021	MET	MET mutant NSCLC
Sotorasib	2021	KRAS	KRAS G12C mutant NSCLC
Amivantamab	2021	EGFR and MET	EGFR ex20ins NSCLC
Adagrasib	2022	KRAS	KRAS G12C mutant NSCLC
Trastuzumab	2022	HER2	HER2 mutant NSCLC
Binimetinib	2023	BRAF	BRAF mutant NSCLC
Encorafenib	2023	BRAF	BRAF mutant NSCLC
Repotrectinib	2023	ROS1	ROS1 positive NSCLC

## 2 Biomarkers and target therapies

### 2.1 EGFR inhibitors

**Figure 1:**
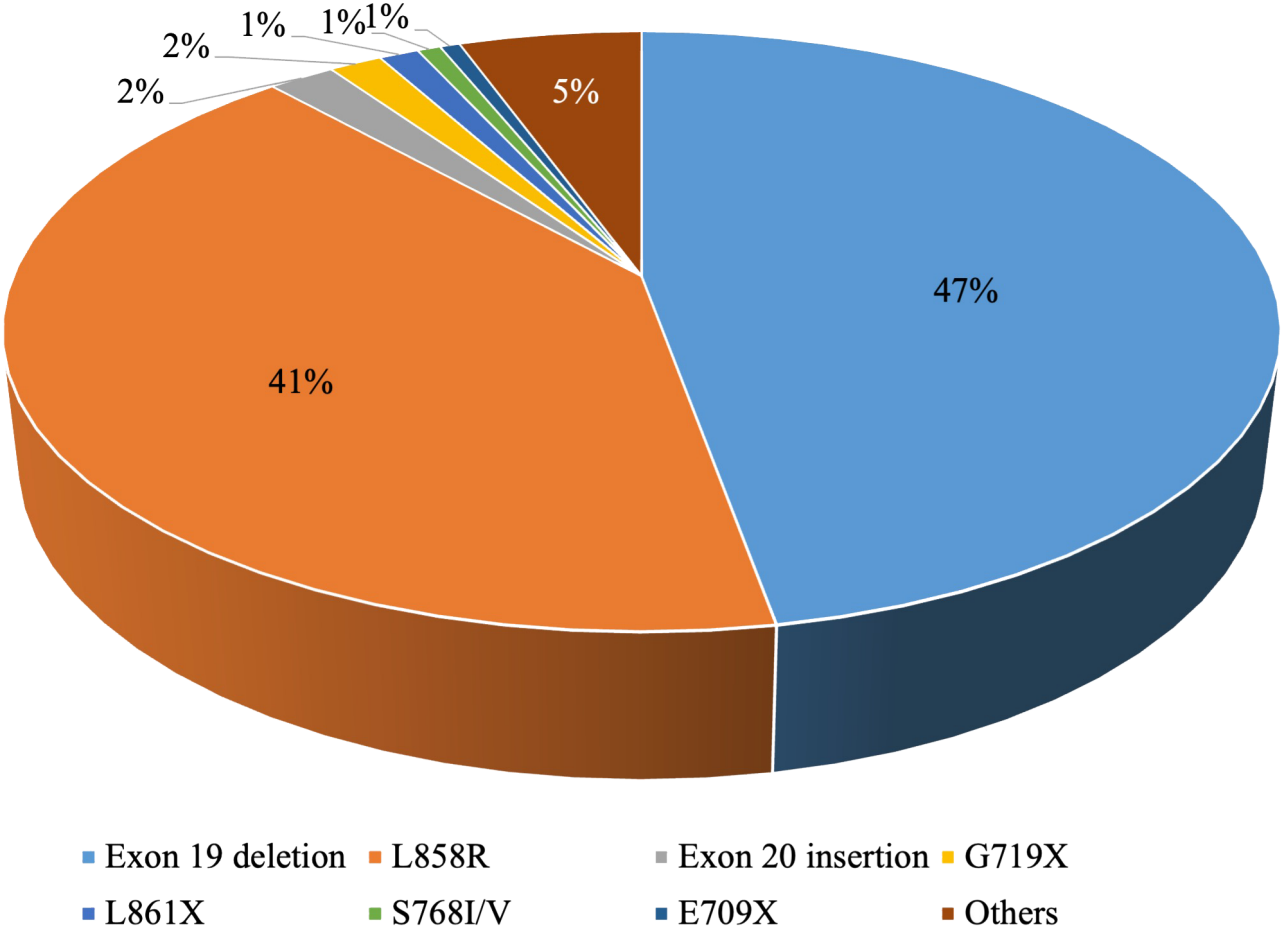
The frequencies of EGFR mutations in NSCLC.

EGFR is the most common driver gene in NSCLC. The mutation frequency is approximately 10-15% in Western Europe and North America and can be as high as 30%-50% in East Asia (9, 10). Common *EGFR* mutations involve exon 19 deletions and the exon 21 mutation p.L858R, while less frequent mutations include p.S768I/V, p.L861X, and p.G719X (11) (**Figure 1**).

#### 2.1.1 First-generation medications

Gefitinib and erlotinib were both reversible inhibitors of the first-generation EGFR TKIs. They can selectively and reversibly prevent ATP binding, thereby inhibiting EGFR autophosphorylation (12). An analysis of five clinical studies in which erlotinib or gefitinib was used as first-line treatment in NSCLC (stage IIIB or IV) revealed that the response rate was 67% in patients with sensitizing *EGFR* mutations (13).

##### 2.1.1.1 Erlotinib

Erlotinib has shown better efficacy than conventional chemotherapy in advanced NSCLC patients with *EGFR* mutations in multiple randomized phase III trials. In the EURTAC trial, patients receiving erlotinib demonstrated a response rate of 58% with a median PFS of 9.7 months, whereas those receiving conventional chemotherapy exhibited a response rate of 15% with a median PFS of 5.2 months (14). In the trial CALGB30406, erlotinib monotherapy achieved an impressive response rate of 70% (15). Another phase III trial reported a higher objective response rate in the gefitinib group compared to the chemotherapy group (73.7% vs. 30.7%) (16).

##### 2.1.1.2 Gefitinib

The phase III randomized trial IPASS evaluated the efficacy of gefitinib in previously untreated NSCLC patients in East Asia, patients treated with gefitinib exhibited a significantly high objective response rate of 71.2% compared to those treated with carboplatin–paclitaxel (17). The OPTIMAL trial also reported a superior response rate in the gefitinib group compared to the chemotherapy group (83% vs. 36%) (18). The phase III randomized trial WJOG5108L reported similar response rates for gefitinib and erlotinib at 55.0% and 58.9%, respectively (19).

#### 2.1.2 Second-generation medications

##### 2.1.2.1 Afatinib

Afatinib, a second-generation oral TKI, exerts irreversible inhibition targeting the ErbB/HER receptor family including EGFR and HER2 (20). In a phase IIB trial comparing afatinib and gefitinib for first-line treatment in common *EGFR* mutation metastatic adenocarcinoma patients, afatinib demonstrated a significantly higher objective tumor response rate compared to gefitinib (70% vs. 56%) (21). Updated results revealed no significant difference in OS between the two groups (22). A subgroup analysis of several LUX-LUNG trials (LUX-LUNG 2, 3, and 6) evaluated the efficacy of afatinib in patients with mutation-positive metastatic NSCLC. The response rate was 77.8% in patients with *EGFR* p.G719X mutation, 100% in p.S768I, and 56.3% in p.L861Q (23). Notably, these findings should be interpreted cautiously as treatment crossover occurred in most patients (72% in LUX-LUNG 3 and 80% in LUX-LUNG 6).

##### 2.1.2.2 Dacomitinib

Dacomitinib is a second-generation oral TKI, that exerts irreversible inhibition on ErbB/HER receptors, including *EGFR*, *HER1*, *HER2*, and *HER4*.In the phase III randomized trial ARCHER1050, patients receiving dacomitinib as first-line treatment exhibited an objective response rate of 75% (24). Subsequent updated data indicated that dacomitinib-treated patients experienced longer progression-free survival (PFS) (14.7 months vs. 9.2 months) and OS (34.1 months vs. 27 months) compared to the gefitinib group (25, 26).

#### 2.1.3 Third-generation medications

Approximately 60% of patients who progressed on first- and second-generation EGFR TKI treatment harbor EGFR p.T790M mutation. The third-generation EGFR TKIs were originally designed to overcome the resistance caused by acquired EGFR p.T790M mutation. Osimertinib, an oral and irreversible TKI, exhibits selectivity for both common *EGFR* mutations and p.T790M mutation, with activity within the central nervous system (CNS) (27–29). Osimertinib is the first third-generation EGFR-TKI approved by the Food and Drug Administration (FDA) and the European Medicines Agency (EMA) for metastatic NSCLC patients with *EGFR* p.T790M mutation (30).

In a phase III randomized trial (AURA3), involving patients with EGFR p.T790M-positive metastatic NSCLC progressing after first-line treatment, the objective response rate was significantly superior with osimertinib (71%) compared to chemotherapy (31%). Osimertinib also exhibited a longer PFS (10.1 vs. 4.4 months). Notably, in the subgroup of patients with CNS metastases, osimertinib presented a prolonged PFS compared to those treated with platinum–pemetrexed (8.5 vs. 4.2 months) (31). The BLOOM study which increased the standard dose of osimertinib from 80 mg once daily to 160 mg once daily, have proved beneficial of the higher dose of osimertinib for patients with leptomeningeal disease progression with EGFR mutations, irrespective of p.T790M status, with an objective response rate of 62% (32, 33).

In addition to the second- or third-line use to overcome resistance of first- and second-generation EGFR TKI treatment, osimertinib has been used as first-line to treat EGFR mutant NSCLC patients. A Multicenter, Phase II Trial (KCSG-LU15-09) demonstrated an objective response rate of 50% for osimertinib as first-line treatment in 37 patients with EGFR rare mutations, including p.S768I, p.L861Q, and p.G719X (34). The phase III randomized trial (FLAURA) also proved a longer median OS with osimertinib as first-line treatment than with erlotinib or gefitinib (38.6 months vs. 31.8 months), though the objective response rate was comparable (80% vs 76%) (35, 36).

#### 2.1.4 Other medications

##### 2.1.4.1 Amivantamab

Amivantamab is a bispecific human antibody to both EGFR and MET receptors that bypasses resistance to EGFR TKIs (37). CHRYSALIS study, a phase I study, evaluated the efficacy of Amivantamab-vmjw as a subsequent treatment in 81 metastatic NSCLC patients with *EGFR* exon 20 insertion. The overall response rate reported in this cohort was 40% (37). In a phase III study (PAPILLON), amivantamab-chemotherapy significantly improved PFS of patients with EGFR exon 20 insertions who had not received previous systemic therapy when compared to chemotherapy alone (median, 11.4 months and 6.7 months, respectively) (38). And MARIPOSA evaluated the therapeutic efficacy of Amivantamab plus carboplatin-pemetrexed (chemotherapy) with and without Lazertinib in patients with EGFR-mutated (exon 19 deletions or L858R) locally advanced or metastatic NSCLC after disease progression on Osimertinib. The median PFS was significantly longer for amivantamab-chemotherapy and amivantamab-lazertinib-chemotherapy versus chemotherapy (6.3 and 8.3 versus 4.2 months, respectively) (39).

##### 2.1.4.2 Mobocertinib

Mobocertinib is an oral TKI selectively inhibiting *EGFR* and *HER2* exon 20 insertion mutations (40, 41). A phase I/II study evaluated the efficacy of mobocertinib as a subsequent treatment in patients with *EGFR* exon 20 insertion mutation. The objective response rate was 28%, with a median duration of response of 17.5 months and a median PFS of 7.3 months (40). Subsequently, mobocertinib received FDA accelerated approval for advanced or metastatic NSCLC in adults with EGFR exon 20 insertion mutations who progressed during or after platinum-based chemotherapy.

However, results from the phase III trial, EXCLAIM-2, indicated that the objective response rates and disease control rates between the mobocertinib and chemotherapy groups are similar (response rate: 32% vs. 30%, control rate: 87% vs. 80%) (42). As a result, the FDA and Takeda withdrew mobocertinib in America in October 2023, as it did not meet the primary endpoint of the study.

##### 2.1.4.3 Cetuximab

Cetuximab is a monoclonal antibody to EGFR. In a large phase III randomized trial, FLEX, the combination of chemotherapy and cetuximab proved higher overall response rates than chemotherapy alone (36% vs. 29%) and comparable median OS (11.3 vs. 10.1 months) (43). However, this combination exhibited poorer tolerability considering the nearly 40% incidence of grade 4 neutropenia. Therefore, the use of cetuximab is not yet recommended in NSCLC.

### 2.2 ALK inhibitors

*ALK* gene rearrangements occurred in approximately 3-5% of NSCLC patients (44). So far, more than 19 distinct *ALK* fusion partners have been identified in NSCLC, including *EML4*, *KIF5B*, *KLC1*, and *TPR* (45). The most common fusion was *EML4::ALK*, existing in about 85% of *ALK*-rearrangement NSCLC.

#### 2.2.1 First-generation medications

##### 2.2.1.1 Crizotinib

Crizotinib is a first-generation oral TKI and the first TKI approved for treating ALK-positive NSCLC, effectively inhibits *ALK* rearrangements, *ROS1* rearrangements, high-level *MET* amplification, and METex14 skipping mutations. In phase I and II studies, crizotinib demonstrated objective tumor responses in approximately 60% of *ALK*-positive NSCLC patients, with a median PFS ranging from 7 to 10 months (46–48). A phase III randomized study, PROFILE 1014, assessing the efficacy of crizotinib as first-line targeted therapy, yielded promising results with an objective response rate of 74% (49). For *ALK*-positive patients progressing after first-line chemotherapy, crizotinib has shown efficacy in improving PFS (7.7 months) and enhancing response rates (65%) (50).

#### 2.2.2 Second-generation medications

##### 2.2.2.1 Alectinib

Alectinib is a selective second-generation oral ALK inhibitor with high CNS penetration. It has demonstrated activity against several secondary mutations associated with acquired resistance to crizotinib, such as p.T1151L, p.1152insT, p.L1196M, p.C1156Y, p.F1174L, and p.G1269A (51, 52).

The ALEX trial, a phase III randomized study, compared the efficacy of alectinib and crizotinib as first-line treatments in 303 ALK-positive advanced NSCLC patients, including those with asymptomatic brain metastases. The response rate in the alectinib group was 82.9% and 75.5% in the crizotinib group (53). Another phase III trial, J-ALEX, enrolled 207 ALK inhibitor-naive Japanese patients with ALK-positive NSCLC, also proved that alectinib as a first-line treatment achieved a higher objective response rate compared to crizotinib (92% vs. 79%) (54).

Efficacy of alectinib as subsequent treatments was reported by phase II trials with a total response rate of 48% to 50% in metastatic NSCLC patients with *ALK* rearrangement progressing after crizotinib treatment (55, 56).

##### 2.2.2.2 Brigatinib

Brigatinib is a second-generation TKI that inhibits a broad spectrum of *ALK* rearrangements. As first-line treatment, brigatinib was reported a higher systemic objective response rate of 71% than crizotinib (60%) in the ALTA-1L trial. The intracranial response rate was also notably higher with brigatinib (78%) compared to crizotinib (29%) (57). Updated data further confirmed that the 3-year PFS in the brigatinib group was superior to crizotinib (43% vs. 19%) (58).

A phase II study, ALTA, evaluated the efficacy of two different doses of brigatinib in *ALK*-positive metastatic NSCLC patients who had experienced disease progression on or intolerance to crizotinib. The overall response rate ranged from 45% to 54%. In patients with measurable brain metastases, the intracranial overall response rate was observed to be between 42% and 67% (59, 60).

##### 2.2.2.3 Ceritinib

Ceritinib is a second-generation oral TKI designed for *ALK* and *ROS1* rearrangements (61), showing promising results in various clinical trials. In the ASCEND-4 trial, the overall response to ceritinib as first-line therapy was 72·5% with a median PFS of 16.6 months, as compared with 26·7% with a median PFS of 8.1 months in the chemotherapy group (62).

As subsequent treatment in patients with prior exposure to at least two treatments, ceritinib was reported an overall response rate of 38.6%, with a concurrent intracranial response rate of 45.0% in a phase II study (ASCEND-2) (63), and a higher overall response rate of 45% than pemetrexed or docetaxel chemotherapy (8%) (64).

#### 2.2.3 Third-generation medications

Lorlatinib, a third-generation oral TKI with excellent CNS penetration, selectively inhibits ALK and ROS1. It exhibits the ability to inhibit *ALK* resistance mutations that emerge following treatment with first and second-generation ALK inhibitors (65–68). In the phase III randomized trial, CROWN, lorlatinib demonstrated complete CNS responses in 61% of patients with baseline brain metastases, compared to only 15% with crizotinib (69). Updated data reveals a lower cumulative CNS progression rate with lorlatinib (7%) than crizotinib (72%) over 12 months, and higher 1-year PFS rates of 78% than 22% for crizotinib in patients with brain metastases (70).

Lorlatinib also remains effective for patients experiencing progression after treatment of other ALK inhibitors, especially those with CNS involvement. Among patients with measurable baseline CNS lesions, 47% achieved objective responses, and 63% achieved an objective intracranial response (66, 67).

### 2.3 BRAF inhibitors

*BRAF* mutations manifest in 1%–5% of NSCLC patients (71–74). The most common mutation is p.V600E, accounting for approximately 50% of *BRAF*-mutated cases (75). Other *BRAF* mutations include p.D594G and p.G469A/V, observed in 35% and 6% of *BRAF*-mutated NSCLC patients, respectively (74). For NSCLC patients with p.V600E mutation, the FDA has currently approved two combinations of RAF and MEK inhibitors: dabrafenib/trametinib and encorafenib/binimetinib.

#### 2.3.1 Dabrafenib/trametinib

In a phase II trial, dabrafenib/trametinib as first-line treatment demonstrated a robust overall response rate of 64% in 36 patients with *BRAF* p.V600E mutation (76). An updated analysis of this trial revealed a 5-year OS rate of 22% (1). Another dual-cohort phase II study conducted a comparative analysis between patients receiving dabrafenib monotherapy and combination therapy with dabrafenib and trametinib. The results indicated a distinct overall response rate of 33% and 67%, and median PFS durations of 5.5 months and 10.2 months, respectively (77).

#### 2.3.2 Encorafenib/binimetinib

In the PHAROS trial, an open-label, multicenter, single-arm study, an impressive overall response rate of 75% was observed among the 59 treatment-naive patients with *BRAF* p.V600E mutation, with a median duration of response not achieved. In the cohort of 39 previously treated patients, the overall response rate was 46%, and the median duration of response was 16.7 months (78).

### 2.4 ERBB2 (HER2) inhibitors

#### 2.4.1 Ado-trastuzumab emtansine

Ado-Trastuzumab Emtansine, also known as T-DM1, is a humanized antibody-drug conjugate comprising the HER2-targeting antibody trastuzumab and the microtubule inhibitor emtansine (79). In a phase II basket trial, the efficacy of ado-trastuzumab emtansine was assessed in patients with metastatic NSCLC and *HER2* mutations, revealing a partial response rate of 44% (79, 80). Another study focused on patients with *HER2* exon20 insertion mutations, indicating an objective response rate of 38% with ado-trastuzumab emtansine (81). These findings underscore the potential of ado-trastuzumab emtansine as a targeted therapeutic option for patients with *HER2*-mutated NSCLC.

#### 2.4.2 Fam-trastuzumab deruxtecan-nxki

Fam-trastuzumab deruxtecan-nxki, a humanized monoclonal antibody-drug conjugate comprising trastuzumab linked to deruxtecan, is a topoisomerase I inhibitor (82). A phase I trial investigated the efficacy of fam-trastuzumab deruxtecan-nxki in *HER2*-mutant NSCLC patients, representing an objective response rate of 72.7% (83). The DESTINY-Lung01, a phase II study, revealed an objective response rate of 55% in 91 patients treated with fam-trastuzumab deruxtecan-nxki (82).

### 2.5 KRAS inhibitors

*KRAS* mutations are identified in approximately 30% of NSCLC patients (84). These mutations are predominantly (>95%) located at codons 12 and 13. The p.G12C variant was the most prevalent, constituting 39% of all *KRAS* mutations, followed by p.G12V (21%) and p.G12D (17%) variants (85). Sotorasib and adagrasib are both an oral inhibitor to the RAS GTPase family, demonstrating efficacy in inhibiting the *KRAS* p.G12C mutation in patients with metastatic NSCLC who have previously undergone chemotherapy (± immunotherapy).

#### 2.5.1 Sotorasib

Sotorasib, as a small-molecule inhibitor, irreversibly binds to the non-active GDP pocket of KRAS, forming an irreversible covalent bond with the cysteine residue in *KRAS* p.G12C. This covalent interaction locks the protein in an inactive state. By disrupting the KRAS signaling pathway, sotorasib inhibits cell growth as well as tumor progression both *in vitro* and *in vivo* and induces apoptosis in *KRAS* p.G12C tumor cell lines (86, 87).

In a phase II study involving 126 patients with *KRAS* p.G12C-positive advanced NSCLC who had prior platinum-based chemotherapy (with or without immunotherapy), subsequent treatment with sotorasib showed a partial response rate of 33.9% and complete response rate of 4.2% (88). The phase III randomized study, CodeBreaK200 trial, has also reported the efficacy of sotorasib in patients in a similar situation (89). Sotorasib demonstrated a significantly higher overall response rate of 28.1% than docetaxel (13.2%). Moreover, the disease-control rate in the sotorasib group was 82.5%, compared to 60.3% in the docetaxel group.

#### 2.5.2 Adagrasib

In a phase II study involving 116 patients who had previously undergone platinum-based chemotherapy with or without immunotherapy, adagrasib demonstrated an objective response rate of 42.9%. The efficacy of adagrasib in cases with *KRAS* mutations beyond p.G12C remains to be systematically evaluated (90).

### 2.6 MET inhibitors

The oncogenic driver genomic alterations associated with MET comprise METex14 skipping mutations and high-level *MET* amplification. High-level *MET* amplification was recently identified as an emerging biomarker. Its definition may vary depending on the reagent kits. When employing Next-Generation Sequencing (NGS), high-level *MET* amplification is defined as the copy number greater than 10 (91). The FDA has not yet approved recommended drugs for NSCLC patients carrying these mutations, despite their approval in other tumor types.

#### 2.6.1 Capmatinib

Capmatinib is an oral TKI selectively targeting *MET* alterations. The GEOMETRY trial revealed that capmatinib achieved an overall response rate of 68% as a first-line treatment, and 41% as subsequent treatment in patients with METex14 skipping mutations. While in patients with high-level *MET* amplification, the response rate was 40% as the first-line therapy, and 29% as subsequent therapy (91). Notably, the updated data of GEOMETRY indicate that capmatinib exhibits anti-tumor efficacy within the brain (92). Another study revealed an overall response rate of 50% in a cohort of 10 patients with high-level *MET* amplification (93).

#### 2.6.2 Crizotinib

Crizotinib is an oral TKI that inhibits METex14 skipping mutation and high-level *MET* amplification. A phase II study evaluated the efficacy of crizotinib in 69 patients with METex14 skipping mutations. The objective response rate was 32%, with a median PFS of 7.3 months (94). The PROFILE1001 study investigated the efficacy of crizotinib in advanced NSCLC patients with varying levels of *MET* amplification. Patients with *MET* genomic copy number over 10 demonstrated an overall response rate of 29% (95).

#### 2.6.3 Tepotinib

Tepotinib is a selective oral TKI that inhibits METex14 skipping mutation and high-level *MET* amplification. A phase II study (VISION) assessed the efficacy of tepotinib in patients with *MET* mutations. The response rate in patients with METex14 skipping mutations was 46%. Another cohort comprising 24 patients with *MET* amplification but lacking METex14 skipping mutations exhibited an overall response rate of 41.7% (96, 97).

### 2.7 NTRK1/2/3 inhibitors

*NTRK1/2/3* gene fusions encode TRK fusion proteins, serving as oncogenic drivers in multiple solid tumors, including lung, thyroid, salivary gland, and sarcoma (98). Entrectinib and larotrectinib are both inhibitors of TRK fusion proteins in unresectable or metastatic solid tumors.

#### 2.7.1 Entrectinib

The efficacy of entrectinib was evaluated in three phase I or II trials (STARTRK-2, STARTRK-1, ALKA-372-001). A pooled analysis revealed an overall response rate of 70% in 10 *NTRK* gene fusion-positive NSCLC patients treated with entrectinib (99–101).

#### 2.7.2 Larotrectinib

A study comprising 55 patients with various solid tumors and positive *NTRK* gene fusions revealed an overall response rate of 75% with larotrectinib (98). The updated data demonstrated that 90% of patients still remained alive one year after treatment. Furthermore, among 35 *NTRK* fusion cancer patients, the overall response rate reached 74% (102).

### 2.8 RET inhibitors

The *RET* gene is observed in 1-2% of all NSCLC patients with chromosomal rearrangements and is involved in various fusion partners such as KIF5B, TRIM33, CCDC6, and NCOA4 (103, 104).

#### 2.8.1 Pralsetinib

In a phase I/II study (ARROW), pralsetinib was assessed in metastatic NSCLC patients with *RET* rearrangements. The overall response rate of pralsetinib was 70% as a first-line treatment, and 61% as a subsequent treatment reached 61% (105). The FDA approved pralsetinib in 2020 for the treatment of metastatic *RET* fusion-positive NSCLC patients. It is the first oral TKI targeting *RET* fusions (106).

#### 2.8.2 Selpercatinib

A phase I/II study, Libretto-001, along with its updated results, reveals that selpercatinib exhibits remarkable efficacy in NSCLC patients with *RET* rearrangements. The overall response rate for first-line treatment was 85%, while 64% for subsequent treatment. Notably, in patients with brain metastases, selpercatinib demonstrated effectiveness in 91% of cases (107, 108).

#### 2.8.3 Cabozantinib

In a prospective phase II trial involving 26 *RET* fusion-positive patients treated with cabozantinib, the overall response rate was 28% (109, 110).

### 2.9 ROS1 inhibitors

#### 2.9.1 Crizotinib

Crizotinib is a multitargeted inhibitor targeting MET, ALK, and ROS1. In an early-phase study, crizotinib demonstrated considerable efficacy in *ROS1*-rearranged NSCLC (111). The objective response rate in the expansion cohort treated with crizotinib reached 72%. The overall response duration was 17.6 months, with a median PFS of 19.2 months (112).

Three phase II studies confirmed an overall response rate of more than 70% with crizotinib in patients with *ROS1* rearrangement. A phase II trial evaluating the efficacy of crizotinib in 127 East Asian patients reported an overall response rate of 72% (113). The PROFILE 1001 study and updated data reported an objective response rate of 72% in 53 ROS1-positive advanced NSCLC patients, including 3 complete responses and 33 partial responses (4, 114). The multicenter trial, EUCROSS study, reported a total response rate of 70% in 30 patients treated with crizotinib (115). Additionally, a retrospective study assessing crizotinib in stage IV *ROS1*-rearranged NSCLC patients (n=30) reported an overall response rate of 80%, with a median PFS of 9.1 months (116).

#### 2.9.2 Lorlatinib

Lorlatinib is an oral third-generation TKI targeting both ALK and ROS1 with significant CNS penetration. It was evaluated in a phase I/II trial for its efficacy in ROS1-positive metastatic NSCLC patients. The objective response rate in patients previously treated with crizotinib reached 35%, while treatment-naive patients demonstrated a 62% objective response rate. Notably, intracranial responses were observed in 50% of patients with prior crizotinib treatment and 64% of treatment-naive patients (65).

#### 2.9.3 Entrectinib

Entrectinib is an oral TKI inhibiting multiple tyrosine kinases, including ROS1 and TRK. A pooled analysis of 53 patients with *ROS1* rearrangement across several phase I and II trials (STARTRK-2 trial, STARTRK-1 trial, ALKA-372-001 trial) who received entrectinib as first-line treatment demonstrated an overall response rate of 77%, with a 55% intracranial response rate (100, 101, 117). Although entrectinib exhibits superior CNS penetration compared to crizotinib, it comes with higher toxicity, with an incidence of grade 3 or 4 adverse events of 34% (117).

#### 2.9.4 Ceritinib

Ceritinib is a second-generation oral TKI inhibiting *ALK* and *ROS1* rearrangements. In a phase II trial assessing ceritinib as first-line treatment in *ROS1*-rearranged NSCLC patients (28 evaluable patients), the reported overall response rate was 62%, with 1 case of complete response and 19 cases of partial responses (61).

#### 2.9.5 Repotrectinib

A phase I/II trial assessed the efficacy and safety of repotrectinib in patients with advanced *ROS1* fusion-positive NSCLC. The confirmed overall response rate was 79% among ROS1 TKI-naive patients and 38% among patients previously treated with other ROS1 inhibitors. Notably, responses were observed in intracranial lesions in patients with measurable CNS metastases, as well as in those with resistance mutations following TKI therapy (118).

### 2.10 VEGF or VEGF receptors inhibitors

#### 2.10.1 Bevacizumab

Bevacizumab is a recombinant monoclonal antibody targeting VEGF. In a phase III randomized trial, ECOG4599, involving relapsed or advanced non-squamous NSCLC, the corresponding response rates were 35% in patients treated with a combination of bevacizumab chemotherapy and 15% in those treated with chemotherapy alone (119). Another phase III trial, NEJ026, compared the efficacy of erlotinib combined with bevacizumab to erlotinib monotherapy as first-line treatments in *EGFR*-positive advanced non-squamous NSCLC patients. The objective response rates were similar (erlotinib/Ramucirumab: 72% vs. erlotinib monotherapy: 67%) (120).

#### 2.10.2 Ramucirumab

Ramucirumab is a recombinant monoclonal antibody targeting VEGF receptors. In the phase III randomized trial RELAY, first-line treatment with erlotinib/ramucirumab was compared to erlotinib monotherapy in *EGFR*-mutated advanced NSCLC patients. The overall response rates were similar (erlotinib/ramucirumab: 76% vs. erlotinib monotherapy: 75%) (121). The REVEL trial, a phase III randomized study in metastatic NSCLC patients who experienced disease progression, evaluated the efficacy of ramucirumab/docetaxel compared to docetaxel alone as subsequent therapy. The ramucirumab/docetaxel group exhibited higher overall response rates (23% vs. 14%) and disease control rates (64% vs. 53%) (122).

#### 2.10.3 Nintedanib

Nintedanib is a potent, oral angiokinase inhibitor that targets the pro-angiogenic pathways mediated by VEGFR1-3 (123). In the phase III randomized controlled trial LUME-Lung 1, 1314 stage IIIB/IV patients progressing after first-line chemotherapy were randomly assigned to receive docetaxel plus nintedanib (n=655) or docetaxel plus placebo therapy (n=659). PFS was significantly improved in the nintedanib plus docetaxel group when compared to the docetaxel plus placebo group (median 3.4 months vs. 2.7 months) (PMID: (124)).

## 3 Resistance to targeted therapy

### 3.1 Overview of the mechanisms of resistance to targeted therapies

Resistance to targeted therapies is categorized as either primary (intrinsic) or secondary (acquired) (125). Primary resistance describes a *de novo* lack of therapeutic response, while secondary resistance indicates disease progression after the initial response. Despite distinct resistance mechanisms identified in patients with different gene alterations, there are common mechanisms shared among these cohorts (126). The acquired resistance mechanisms can be broadly classified into two categories.

The first category involves the development of additional genetic alterations in the primary oncogenes, activating continued downstream signaling. This is often attributed to secondary mutations in kinase targets or gene amplifications of the kinase itself (127). The second category of resistance development can occur independently of changes in the target gene. This scenario includes upregulation of bypass signaling pathways, histological changes of tumor tissue, or alterations in drug metabolism (128, 129). Moreover, about 14% of small-cell lung cancer can histologically transform into NSCLC, often accompanied by resistance to the original TKI (130, 131).

In 2010, Jackman et al. proposed the criteria of acquired resistance in *EGFR*-mutant NSCLC (132): 1) Patients must have previously received EGFR inhibitor treatment. 2) Patients harbor either tumor-genotyping confirmed typical *EGFR* mutations associated with drug sensitivity, or objective clinical benefit from treatment with an EGFR inhibitor. 3) Patients develop systemic progression while on continuous treatment with gefitinib or erlotinib within the last 30 days. 4) No additional systemic treatment between cessation of EGFR inhibitor and initiation of new therapy.

### 3.2 Resistance to EGFR inhibitors

#### 3.2.1 Primary resistance

Primary resistance to EGFR inhibitors may be partially attributed to differential TKI sensitivity for different *EGFR* mutations. Typical *EGFR* mutations, including exon 19 deletions and p.L858R, are associated with significant sensitivity to TKIs (128). Conversely, exon 20 insertions or duplications, accounting for about 4% of patients with *EGFR* mutations, appear to have resistance to EGFR inhibitors (133).

#### 3.2.2 Acquired resistance

The earliest report of TKI resistance in *EGFR*-mutant NSCLC identified a substitution of threonine for methionine at residue 790 (p.T790M) (134). Subsequent reports confirmed that p.T790M is the most common mutation responsible for TKI resistance, which is identified in approximately 60% of patients who experience disease progression after initial response to first-line EGFR TKIs treatment (125, 134–140).

Threonine 790 serves as the “gatekeeper” residue, crucial for inhibitor specificity in the ATP binding pocket. The p.T790M mutation activates wild-type (WT) EGFR, introducing an increase in the ATP affinity of the p.L858R mutant by more than an order of magnitude. This is the main mechanism by which the p.T790M mutation confers TKI resistance, reducing the efficacy of any ATP-competitive kinase inhibitor. Irreversible inhibitors can simply overcome this resistance through covalent binding rather than alternative binding (141). Therefore, in patients with *EGFR* p.T790M-positive metastatic NSCLC experiencing progression after first-line treatment, osimertinib as an irreversible EGFR-TKI can achieve an objective response rate of over 70% (31).

Other secondary mutations include p.D761Y, p.L747S, and p.T854A. They reduce the sensitivity to EGFR inhibitors, but the resistance mechanism remains unknown (142). In the AURA trial, the acquired p.C797S mutation was observed in 14% of the samples (31). The p.C797S mutation frequency was 7% when osimertinib was used as first-line therapy (35). The *EGFR* p.C797S mutation, in which cysteine at codon 797 is replaced by serine in the ATP-binding site, results in the loss of the covalent bond between osimertinib and mutated *EGFR*. Predictably, the p.C797S mutation also leads to cross-resistance by preventing other irreversible third-generation TKIs from binding to the EGFR active site (143–145).

TKI resistance may also activate bypass signaling pathways, such as *MET* amplification (15-19%), *PIK3CA* mutations (6-7%), *KRAS* mutations (3%), and *HER2* amplification (2-5%) (146, 147). Bypass pathway activation leads to TKI resistance by sustaining activation of EGFR downstream signaling pathways.

### 3.3 Resistance to ALK inhibitors

The primary resistance to ALK inhibitors may be due to the different sensitivity of *EML4::ALK* variants and other *ALK* fusion genes to ALK inhibitors (148). Acquired resistance to ALK inhibitors typically occurs within the first year of treatment (125). Secondary mutations in the enzyme are the common mechanism of TKI resistance. It is noteworthy that multiple secondary mutations can occur in ALK-positive patients upon TKI resistance. The first “gatekeeper” mutation identified in the *EML4::ALK* kinase domain is p.L1196M (149). The substitution of leucine for methionine at position 1196 in the ATP binding pocket generates a mutated large amino acid side chain, which hinders crizotinib from binding to its receptor. Other identified acquired resistance point mutations include p.G1128A, p.1151Tins, p.L1152P/R, p.C1156Y, p.I1171T/N/S, p.F1174V, p.V1180L, p.G1202R, p.S1206Y/C, p.E1210K, and p.G1269A (150–155).

Numerous studies suggest that second-generation drugs such as alectinib, ceritinib, brigatinib, and ensatinib may be more effective than chemotherapy when treating NSCLC patients with no response to first-generation ALK inhibitors (64, 156–158). In patients treated with second-generation ALK inhibitors, the p.G1202R mutation is the most common secondary ALK mutation, appearing in 21% of ceritinib-treated patients, 29% of alectinib-treated patients, and 43% of brigatinib-treated patients (159).

A gain in *ALK* gene fusion copy number (more than two-fold increase) has recently been proposed as a mechanism of resistance to crizotinib in both *in vitro* and in patients (150, 155). Based on single circulating tumor cell sequencing, another study reported repeated mutations in the RTK-KRAS (*EGFR*, *KRAS*, *BRAF* genes), TP53, and other genes in the ALK-independent pathway in crizotinib-resistant patients (160).

Resistance to ALK inhibitors can also occur through the activation of bypass signaling pathways, including YAP transcription co-regulator, EGFR signaling, KIT amplification, the IGF-1R pathway, MAPK amplification, the *BRAF* p.V600E mutation, and *MET* amplification (155, 161–165). *MET* amplification was observed in 15% of tumor samples from patients progressing after second-generation ALK inhibitors, and in 12% and 22% of tumor biopsy samples from patients progressing on second-generation inhibitors or lorlatinib, respectively (166).

### 3.4 Resistance to ROS1 inhibitors

Single nucleotide mutations in the ROS1 kinase domain, such as p.D2033N, p.G2032R/K, p.L2026M, p.L2155S, and p.S1986F/Y, have been reported leading to acquired resistance to ROS1 TKIs in *ROS1* fusion-positive NSCLC through preclinical and clinical studies (167–171). These mutations diminish the efficacy of kinase inhibitors (112, 168, 172).

A study evaluating biopsies from 55 patients progressing after TKI treatment found that *ROS1* mutations were identified in 38% of post-crizotinib biopsies and 46% of post-lorlatinib biopsies. Approximately one-third of patients harbored the most common mutation *ROS1* p.G2032R. Additional *ROS1* mutations emerged following crizotinib treatment, including p.D2033N (2.4%), p.S1986F (2.4%), p.L2086F (3.6%), p.G2032R/p.L2086F (3.6%), and p.G2032R/p.S1986F/p.L2086F (3.6%). p.S1986F/p.L2000V (3.6%) was detected in 3.6% of patients receiving lorlatinib treatment (170).

The p.D2033N mutation causes the substitution of aspartate for asparagine at position 2033 in the ROS1 kinase hinge region, thus leading to significant resistance to ROS1 inhibitors *in vitro* (173, 174). The p.L2026M and p.G2032R mutations in the ROS1 kinase domain confer crizotinib resistance by altering the “gatekeeper” position of ROS1 inhibitor binding (168, 175). Additionally, p.S1986F/Y in the kinase domain disrupts crucial activation sites, thereby increasing kinase activity. p.L2155S is anticipated to confer crizotinib resistance through protein dysfunction (176).

The mutations and/or copy number increases of genes in other RTKs or downstream MAPK pathway are also involved in the mechanism of resistance to ROS1 inhibitor (177). Mediators involved in this pathway include KRAS, NRAS, EGFR, HER2, MET, KIT, BRAF, and MEK, either as downstream or bypass mediators (167, 168, 172, 174). *KRAS* p.G12D and *BRAF* p.V600E mutations are associated with crizotinib treatment, while *NRAS* p.Q61K is associated with entrectinib treatment (178).

## 4 Strategies for overcoming resistance to TKIs

Targeted therapies have significantly improved the prognosis of NSCLC patients with relevant genetic alterations, which is a major progress in the history of NSCLC treatment. However, part of the patients acquires TKI resistance and disease progression shortly after initial remission. Strategies have been investigated to overcoming resistance to TKIs, which include the continuation of TKI therapy beyond disease progression, combination with other TKIs, and the use of immune checkpoint inhibitors.

### 4.1 Continuation of TKI therapy beyond disease progression

A phase II open-label single-arm trial named ASPIRATION reported that the post-progression erlotinib patients exhibited deeper responses, longer PFS, prolonged time from overall response to progression, and fewer new lung lesions (179). A retrospective analysis of 414 ALK-positive NSCLC patients enrolled in PROFILE 1001 and PROFILE 1005 showed that continuation of crizotinib (>3 weeks) after progression conferred extended progression time and longer OS (180). However, more evidence supports the timely detection of potential resistance mutations and prompt switching to sensitive targeted therapies after disease progression.

### 4.2 Combination with other TKIs

In a phase Ib/II single-arm trial, 47% of EGFR TKI-resistant NSCLC patients with MET gene amplification and 32% of EGFR TKI-resistant patients with MET overexpression responded to the MET inhibitor capmatinib in combination with EGFR TKI (181). In another phase 1b study of the combination of the MET inhibitors savolitinib and gefitinib, up to 52% of patients with EGFR TKI-resistant NSCLC with MET gene amplification had an objective response to the combination treatment regimen (182). In the subsequent INSIGHT study, 67% of EGFR TKI-resistant NSCLC patients with MET gene amplification had an objective therapeutic response to treatment with the MET inhibitor tepotinib combined with gefitinib (183). And in the phase Ib trial of the TATTON study, 64% of NSCLC patients who were resistant to first- or second-generation EGFR TKIs and had MET gene amplification showed improved response to savolitinib combined with osimertinib. However, only 30% of patients who were resistant to third-generation EGFR TKIs and had MET gene amplification showed an objective response to this combination therapy (184).

### 4.3 Immune checkpoint inhibitors

In recent years, checkpoint inhibitor antibodies, including programmed cell death protein 1 (PD-1) inhibitors and programmed death ligand 1 (PD-L1) inhibitors, have demonstrated favorable outcomes in NSCLC treatment by blocking the PD-1 and PD-L1 interaction and enhancing the antitumor effects of endogenous T cells. Pembrolizumab, atezolizumab, and cemiplimab have all been reported to prolong PFS and OS in eligible patients (185–189). However, the efficacy of checkpoint inhibitor antibodies depends on the expression level of PD-L1, and for certain mutations such as *EGFR* exon 19 deletions, *EGFR* p.L858R mutations, or *ALK* rearrangements, they appeared to be less effective (190–194).

In conclusion, targeted therapy has brought significant benefits to NSCLC patients, but the emergence of TKI resistance poses a formidable obstacle. The treatment of NSCLC still has a long way to go.

## Author contributions

HZ: Writing – original draft, Writing – review & editing. YZhang: Writing – original draft, Writing – review & editing. YZhu: Visualization, Writing – review & editing. TD: Writing – review & editing. ZL: Funding acquisition, Supervision, Writing – review & editing.

## References

[1] Planchard D Besse B Groen HJM Hashemi SMS Mazieres J Kim TM. Phase 2 study of dabrafenib plus trametinib in patients with BRAF V600E-mutant metastatic NSCLC: updated 5-year survival rates and genomic analysis. J Thorac Oncol. (2022) 17:103–15. doi: 10.1016/j.jtho.2021.08.011 34455067

[2] Mok T Camidge DR Gadgeel SM Rosell R Dziadziuszko R Kim DW. Updated overall survival and final progression-free survival data for patients with treatment-naive advanced ALK-positive non-small-cell lung cancer in the ALEX study. Ann Oncol. (2020) 31:1056–64. doi: 10.1016/j.annonc.2020.04.478 32418886

[3] Lin JJ Cardarella S Lydon CA Dahlberg SE Jackman DM Jänne PA. Five-year survival in EGFR-mutant metastatic lung adenocarcinoma treated with EGFR-TKIs. J Thorac Oncol. (2016) 11:556–65. doi: 10.1016/j.jtho.2015.12.103 PMC4979601 26724471

[4] Shaw AT Riely GJ Bang YJ Kim DW Camidge DR Solomon BJ. Crizotinib in ROS1-rearranged advanced non-small-cell lung cancer (NSCLC): updated results, including overall survival, from PROFILE 1001. Ann Oncol. (2019) 30:1121–6. doi: 10.1093/annonc/mdz131 PMC6637370 30980071

[5] Garon EB Hellmann MD Rizvi NA Carcereny E Leighl NB Ahn MJ. Five-year overall survival for patients with advanced non−Small-cell lung cancer treated with pembrolizumab: results from the phase I KEYNOTE-001 study. J Clin Oncol. (2019) 37:2518–27. doi: 10.1200/JCO.19.00934 PMC6768611 31154919

[6] Singhi EK Horn L Sequist LV Heymach J Langer CJ. Advanced non-small cell lung cancer: sequencing agents in the EGFR-mutated/ALK-rearranged populations. Am Soc Clin Oncol Educ Book. (2019) 39:e187–97. doi: 10.1200/EDBK_237821 31099642

[7] Antonia SJ Borghaei H Ramalingam SS Horn L De Castro Carpeño J Pluzanski A. Four-year survival with nivolumab in patients with previously treated advanced non-small-cell lung cancer: a pooled analysis. Lancet Oncol. (2019) 20:1395–408. doi: 10.1016/S1470-2045(19)30407-3 PMC7193685 31422028

[8] Zhao Y Wang S Yang Z Dong Y Wang Y Zhang L. Co-occurring potentially actionable oncogenic drivers in non-small cell lung cancer. Front Oncol. (2021) 11:665484. doi: 10.3389/fonc.2021.665484 34221980 PMC8242190

[9] Graham RP Treece AL Lindeman NI Vasalos P Shan M Jennings LJ. Worldwide frequency of commonly detected EGFR mutations. Arch Pathol Lab Med. (2018) 142:163–7. doi: 10.5858/arpa.2016-0579-CP 29106293

[10] Han B Tjulandin S Hagiwara K Normanno N Wulandari L Laktionov K. EGFR mutation prevalence in Asia-Pacific and Russian patients with advanced NSCLC of adenocarcinoma and non-adenocarcinoma histology: The IGNITE study. Lung Cancer. (2017) 113:37–44. doi: 10.1016/j.lungcan.2017.08.021 29110846

[11] Harrison PT Vyse S Huang PH. Rare epidermal growth factor receptor (EGFR) mutations in non-small cell lung cancer. Semin Cancer Biol. (2020) 61:167–79. doi: 10.1016/j.semcancer.2019.09.015 PMC7083237 31562956

[12] Martinez-Marti A Navarro A Felip E. Epidermal growth factor receptor first generation tyrosine-kinase inhibitors. Transl Lung Cancer Res. (2019) 8:S235–s246. doi: 10.21037/tlcr.2019.04.20 31857948 PMC6894987

[13] Jackman DM Miller VA Cioffredi LA Yeap BY Jänne PA Riely GJ. Impact of epidermal growth factor receptor and KRAS mutations on clinical outcomes in previously untreated non-small cell lung cancer patients: results of an online tumor registry of clinical trials. Clin Cancer Res. (2009) 15:5267–73. doi: 10.1158/1078-0432.CCR-09-0888 PMC3219530 19671843

[14] Rosell R Carcereny E Gervais R Vergnenegre A Massuti B Felip E. Erlotinib versus standard chemotherapy as first-line treatment for European patients with advanced EGFR mutation-positive non-small-cell lung cancer (EURTAC): a multicentre, open-label, randomised phase 3 trial. Lancet Oncol. (2012) 13:239–46. doi: 10.1016/S1470-2045(11)70393-X 22285168

[15] Jänne PA Wang X Socinski MA Crawford J Stinchcombe TE Gu L. Randomized phase II trial of erlotinib alone or with carboplatin and paclitaxel in patients who were never or light former smokers with advanced lung adenocarcinoma: CALGB 30406 trial. J Clin Oncol. (2012) 30:2063–9. doi: 10.1200/JCO.2011.40.1315 PMC3397694 22547605

[16] Maemondo M Inoue A Kobayashi K Sugawara S Oizumi S Isobe H. Gefitinib or chemotherapy for non-small-cell lung cancer with mutated EGFR. N Engl J Med. (2010) 362:2380–8. doi: 10.1056/NEJMoa0909530 20573926

[17] Mok TS Wu YL Thongprasert S Yang CH Chu DT Saijo N. Gefitinib or carboplatin-paclitaxel in pulmonary adenocarcinoma. N Engl J Med. (2009) 361:947–57. doi: 10.1056/NEJMoa0810699 19692680

[18] Zhou C Wu YL Chen G Feng J Liu XQ Wang C. Erlotinib versus chemotherapy as first-line treatment for patients with advanced EGFR mutation-positive non-small-cell lung cancer (OPTIMAL, CTONG-0802): a multicentre, open-label, randomised, phase 3 study. Lancet Oncol. (2011) 12:735–42. doi: 10.1016/S1470-2045(11)70184-X 21783417

[19] Urata Y Katakami N Morita S Kaji R Yoshioka H Seto T. Randomized phase III study comparing gefitinib with erlotinib in patients with previously treated advanced lung adenocarcinoma: WJOG 5108L. J Clin Oncol. (2016) 34:3248–57. doi: 10.1200/JCO.2015.63.4154 27022112

[20] Nelson V Ziehr J Agulnik M Johnson M. Afatinib: emerging next-generation tyrosine kinase inhibitor for NSCLC. Onco Targets Ther. (2013) 6:135–43. doi: 10.2147/OTT PMC3594037 23493883

[21] Park K Tan EH O’Byrne K Zhang L Boyer M Mok T. Afatinib versus gefitinib as first-line treatment of patients with EGFR mutation-positive non-small-cell lung cancer (LUX-Lung 7): a phase 2B, open-label, randomised controlled trial. Lancet Oncol. (2016) 17:577–89. doi: 10.1016/S1470-2045(16)30033-X 27083334

[22] Paz-Ares L Tan EH O’Byrne K Zhang L Hirsh V Boyer M. Afatinib versus gefitinib in patients with EGFR mutation-positive advanced non-small-cell lung cancer: overall survival data from the phase IIb LUX-Lung 7 trial. Ann Oncol. (2017) 28:270–7. doi: 10.1093/annonc/mdw611 PMC5391700 28426106

[23] Yang JC Sequist LV Geater SL Tsai CM Mok TS Schuler M. Clinical activity of afatinib in patients with advanced non-small-cell lung cancer harbouring uncommon EGFR mutations: a combined *post-hoc* analysis of LUX-Lung 2, LUX-Lung 3, and LUX-Lung 6. Lancet Oncol. (2015) 16:830–8. doi: 10.1016/S1470-2045(15)00026-1 26051236

[24] Wu YL Cheng Y Zhou X Lee KH Nakagawa K Niho S. Dacomitinib versus gefitinib as first-line treatment for patients with EGFR-mutation-positive non-small-cell lung cancer (ARCHER 1050): a randomised, open-label, phase 3 trial. Lancet Oncol. (2017) 18:1454–66. doi: 10.1016/S1470-2045(17)30608-3 28958502

[25] Mok TS Cheng Y Zhou X Lee KH Nakagawa K Niho S. Improvement in overall survival in a randomized study that compared dacomitinib with gefitinib in patients with advanced non-small-cell lung cancer and EGFR-activating mutations. J Clin Oncol. (2018) 36:2244–50. doi: 10.1200/JCO.2018.78.7994 29864379

[26] Mok TS Cheng Y Zhou X Lee KH Nakagawa K Niho S. Updated overall survival in a randomized study comparing dacomitinib with gefitinib as first-line treatment in patients with advanced non-small-cell lung cancer and EGFR-activating mutations. Drugs. (2021) 81:257–66. doi: 10.1007/s40265-020-01441-6 PMC7932969 33331989

[27] Cross DA Ashton SE Ghiorghiu S Eberlein C Nebhan CA Spitzler PJ. overcomes T790M-mediated resistance to EGFR inhibitors in lung cancer. Cancer Discovery. (2014) 4:1046–61. doi: 10.1158/2159-8290.CD-14-0337 PMC4315625 24893891

[28] Ballard P Yates JW Yang Z Kim DW Yang JC Cantarini M. Preclinical comparison of osimertinib with other EGFR-TKIs in EGFR-mutant NSCLC brain metastases models, and early evidence of clinical brain metastases activity. Clin Cancer Res. (2016) 22:5130–40. doi: 10.1158/1078-0432.CCR-16-0399 27435396

[29] Yang JC-H Kim D-W Kim S-W Cho BC Lee J-S Ye X. Osimertinib activity in patients (pts) with leptomeningeal (LM) disease from non-small cell lung cancer (NSCLC): Updated results from BLOOM, a phase I study. J Clin Oncol. (2016) 34. doi: 10.1200/JCO.2016.34.15_suppl.9002

[30] Remon J Steuer CE Ramalingam SS Felip E. Osimertinib and other third-generation EGFR TKI in EGFR-mutant NSCLC patients. Ann Oncol. (2018) 29:i20–7. doi: 10.1093/annonc/mdx704 29462255

[31] Mok TS Wu YL Ahn MJ Garassino MC Kim HR Ramalingam SS. Osimertinib or platinum-pemetrexed in EGFR T790M-positive lung cancer. N Engl J Med. (2017) 376:629–40. doi: 10.1056/NEJMoa1612674 PMC6762027 27959700

[32] Yang JCH Kim SW Kim DW Lee JS Cho BC Ahn JS. Osimertinib in patients with epidermal growth factor receptor mutation-positive non-small-cell lung cancer and leptomeningeal metastases: the BLOOM study. J Clin Oncol. (2020) 38:538–47. doi: 10.1200/JCO.19.00457 PMC7030895 31809241

[33] Yang JC-H Cho BC Kim D-W Kim S-W Lee J-S Su W-C. Osimertinib for patients (pts) with leptomeningeal metastases (LM) from EGFR-mutant non-small cell lung cancer (NSCLC): Updated results from the BLOOM study. J Clin Oncol. (2017). doi: 10.1200/JCO.2017.35.15_suppl.2020

[34] Cho JH Lim SH An HJ Kim KH Park KU Kang EJ. Osimertinib for patients with non-small-cell lung cancer harboring uncommon EGFR mutations: A multicenter, open-label, phase II trial (KCSG-LU15-09). J Clin Oncol. (2020) 38:488–95. doi: 10.1200/JCO.19.00931 PMC7098834 31825714

[35] Ramalingam SS Vansteenkiste J Planchard D Cho BC Gray JE Ohe Y. Overall survival with osimertinib in untreated, EGFR-mutated advanced NSCLC. N Engl J Med. (2020) 382:41–50. doi: 10.1056/NEJMoa1913662 31751012

[36] Soria JC Ohe Y Vansteenkiste J Reungwetwattana T Chewaskulyong B Lee KH. Osimertinib in untreated EGFR-mutated advanced non-small-cell lung cancer. N Engl J Med. (2018) 378:113–25. doi: 10.1056/NEJMoa1713137 29151359

[37] Park K Haura EB Leighl NB Mitchell P Shu CA Girard N. Amivantamab in EGFR exon 20 insertion-mutated non-small-cell lung cancer progressing on platinum chemotherapy: initial results from the CHRYSALIS phase I study. J Clin Oncol. (2021) 39:3391–402. doi: 10.1200/JCO.21.00662 PMC8791812 34339292

[38] Zhou C Tang KJ Cho BC Liu B Paz-Ares L Cheng S. Amivantamab plus chemotherapy in NSCLC with EGFR exon 20 insertions. N Engl J Med. (2023) 389:2039–51. doi: 10.1056/NEJMoa2306441 37870976

[39] Passaro A Wang J Wang Y Lee SH Melosky B Shih JY. Amivantamab plus chemotherapy with and without lazertinib in EGFR-mutant advanced NSCLC after disease progression on osimertinib: primary results from the phase III MARIPOSA-2 study. Ann Oncol. (2024) 35:77–90. doi: 10.1016/j.annonc.2023.10.117 37879444

[40] Zhou C Ramalingam SS Kim TM Kim SW Yang JC Riely GJ. Treatment outcomes and safety of mobocertinib in platinum-pretreated patients with EGFR exon 20 insertion-positive metastatic non-small cell lung cancer: A phase 1/2 open-label nonrandomized clinical trial. JAMA Oncol. (2021) 7:e214761. doi: 10.1001/jamaoncol.2021.4761 34647988 PMC8517885

[41] Riely GJ Neal JW Camidge DR Spira AI Piotrowska Z Costa DB. Activity and safety of mobocertinib (TAK-788) in previously treated non-small cell lung cancer with EGFR exon 20 insertion mutations from a phase I/II trial. Cancer Discovery. (2021) 11:1688–99. doi: 10.1158/2159-8290.CD-20-1598 PMC8295177 33632775

[42] Janne P Wang B Cho B Zhao J Li J Hochmair M. EXCLAIM-2: Phase III trial of first-line (1L) mobocertinib versus platinum-based chemotherapy in patients (pts) with epidermal growth factor receptor (EGFR) exon 20 insertion (ex20ins)+ locally advanced/metastatic NSCLC. Ann OF Oncol. (2023) 34:S1663–4. doi: 10.1016/j.annonc.2023.10.586

[43] Pirker R Pereira JR Szczesna A von Pawel J Krzakowski M Ramlau R. Cetuximab plus chemotherapy in patients with advanced non-small-cell lung cancer (FLEX): an open-label randomised phase III trial. Lancet. (2009) 373:1525–31. doi: 10.1016/S0140-6736(09)60569-9 19410716

[44] Kohno T Nakaoku T Tsuta K Tsuchihara K Matsumoto S Yoh K. Beyond ALK-RET, ROS1 and other oncogene fusions in lung cancer. Transl Lung Cancer Res. (2015) 4:156–64. doi: 10.3978/j.issn.2218-6751.2014.11.11 PMC4384213 25870798

[45] Zhang SS Nagasaka M Zhu VW Ou SI. Going beneath the tip of the iceberg. Identifying and understanding EML4-ALK variants and TP53 mutations to optimize treatment of ALK fusion positive (ALK+) NSCLC. Lung Cancer. (2021) 158:126–36. doi: 10.1016/j.lungcan.2021.06.012 34175504

[46] Kim D-W Ahn M-J Shi Y De Pas TM Yang P-C Riely GJ. Results of a global phase II study with crizotinib in advanced ALK-positive non-small-cell lung cancer (NSCLC). Ann Oncol. (2012) 23:xi32–3. doi: 10.1016/S0923-7534(20)32006-8

[47] Kwak EL Bang YJ Camidge DR Shaw AT Solomon B Maki RG. Anaplastic lymphoma kinase inhibition in non-small-cell lung cancer. N Engl J Med. (2010) 363:1693–703. doi: 10.1056/NEJMoa1006448 PMC3014291 20979469

[48] Camidge DR Bang YJ Kwak EL Iafrate AJ Varella-Garcia M Fox SB. Activity and safety of crizotinib in patients with ALK-positive non-small-cell lung cancer: updated results from a phase 1 study. Lancet Oncol. (2012) 13:1011–9. doi: 10.1016/S1470-2045(12)70344-3 PMC3936578 22954507

[49] Solomon BJ Mok T Kim DW Wu YL Nakagawa K Mekhail T. First-line crizotinib versus chemotherapy in ALK-positive lung cancer. N Engl J Med. (2014) 371:2167–77. doi: 10.1056/NEJMoa1408440 25470694

[50] Shaw AT Kim DW Nakagawa K Seto T Crinó L Ahn MJ. Crizotinib versus chemotherapy in advanced ALK-positive lung cancer. N Engl J Med. (2013) 368:2385–94. doi: 10.1056/NEJMoa1214886 23724913

[51] Kodama T Tsukaguchi T Yoshida M Kondoh O Sakamoto H. Selective ALK inhibitor alectinib with potent antitumor activity in models of crizotinib resistance. Cancer Lett. (2014) 351:215–21. doi: 10.1016/j.canlet.2014.05.020 24887559

[52] Gadgeel SM Gandhi L Riely GJ Chiappori AA West HL Azada MC. Safety and activity of alectinib against systemic disease and brain metastases in patients with crizotinib-resistant ALK-rearranged non-small-cell lung cancer (AF-002JG): results from the dose-finding portion of a phase 1/2 study. Lancet Oncol. (2014) 15:1119–28. doi: 10.1016/S1470-2045(14)70362-6 25153538

[53] Peters S Camidge DR Shaw AT Gadgeel S Ahn JS Kim DW. Alectinib versus crizotinib in untreated ALK-positive non-small-cell lung cancer. N Engl J Med. (2017) 377:829–38. doi: 10.1056/NEJMoa1704795 28586279

[54] Hida T Nokihara H Kondo M Kim YH Azuma K Seto T. Alectinib versus crizotinib in patients with ALK-positive non-small-cell lung cancer (J-ALEX): an open-label, randomised phase 3 trial. Lancet. (2017) 390:29–39. doi: 10.1016/S0140-6736(17)30565-2 28501140

[55] Ou SH Ahn JS De Petris L Govindan R Yang JC Hughes B. Alectinib in crizotinib-refractory ALK-rearranged non-small-cell lung cancer: A phase II global study. J Clin Oncol. (2016) 34:661–8. doi: 10.1200/JCO.2015.63.9443 26598747

[56] Shaw AT Gandhi L Gadgeel S Riely GJ Cetnar J West H. Alectinib in ALK-positive, crizotinib-resistant, non-small-cell lung cancer: a single-group, multicentre, phase 2 trial. Lancet Oncol. (2016) 17:234–42. doi: 10.1016/S1470-2045(15)00488-X PMC4752892 26708155

[57] Camidge DR Kim HR Ahn MJ Yang JC Han JY Lee JS. Brigatinib versus crizotinib in ALK-positive non-small-cell lung cancer. N Engl J Med. (2018) 379:2027–39. doi: 10.1056/NEJMoa1810171 30280657

[58] Camidge DR Kim HR Ahn MJ Yang JCH Han JY Hochmair MJ. Brigatinib versus crizotinib in ALK inhibitor-naive advanced ALK-positive NSCLC: final results of phase 3 ALTA-1L trial. J Thorac Oncol. (2021) 16:2091–108. doi: 10.1016/j.jtho.2021.07.035 34537440

[59] Kim DW Tiseo M Ahn MJ Reckamp KL Hansen KH Kim SW. Brigatinib in patients with crizotinib-refractory anaplastic lymphoma kinase-positive non-small-cell lung cancer: A randomized, multicenter phase II trial. J Clin Oncol. (2017) 35:2490–8. doi: 10.1200/JCO.2016.71.5904 28475456

[60] Camidge DR Tiseo M Ahn M-J Reckamp K Hansen K Kim S-W. P3. 02a-013 brigatinib in crizotinib-refractory ALK+ NSCLC: central assessment and updates from ALTA, a pivotal randomized phase 2 trial: topic: ALK clinical. J Thorac Oncol. (2017) 12:S1167–9. doi: 10.1016/j.jtho.2016.11.1643

[61] Lim SM Kim HR Lee JS Lee KH Lee YG Min YJ. Open-label, multicenter, phase II study of ceritinib in patients with non-small-cell lung cancer harboring ROS1 rearrangement. J Clin Oncol. (2017) 35:2613–8. doi: 10.1200/JCO.2016.71.3701 28520527

[62] Soria JC Tan DSW Chiari R Wu YL Paz-Ares L Wolf J. First-line ceritinib versus platinum-based chemotherapy in advanced ALK-rearranged non-small-cell lung cancer (ASCEND-4): a randomised, open-label, phase 3 study. Lancet. (2017) 389:917–29. doi: 10.1016/S0140-6736(17)30123-X 28126333

[63] Crinò L Ahn MJ De Marinis F Groen HJ Wakelee H Hida T. Multicenter phase II study of whole-body and intracranial activity with ceritinib in patients with ALK-rearranged non-small-cell lung cancer previously treated with chemotherapy and crizotinib: results from ASCEND-2. J Clin Oncol. (2016) 34:2866–73. doi: 10.1200/jco.2015.65.5936 27432917

[64] Shaw AT Kim TM Crinò L Gridelli C Kiura K Liu G. Ceritinib versus chemotherapy in patients with ALK-rearranged non-small-cell lung cancer previously given chemotherapy and crizotinib (ASCEND-5): a randomised, controlled, open-label, phase 3 trial. Lancet Oncol. (2017) 18:874–86. doi: 10.1016/S1470-2045(17)30339-X 28602779

[65] Shaw AT Solomon BJ Chiari R Riely GJ Besse B Soo RA. Lorlatinib in advanced ROS1-positive non-small-cell lung cancer: a multicentre, open-label, single-arm, phase 1-2 trial. Lancet Oncol. (2019) 20:1691–701. doi: 10.1016/S1470-2045(19)30655-2 31669155

[66] Solomon BJ Besse B Bauer TM Felip E Soo RA Camidge DR. Lorlatinib in patients with ALK-positive non-small-cell lung cancer: results from a global phase 2 study. Lancet Oncol. (2018) 19:1654–67. doi: 10.1016/S1470-2045(18)30649-1 30413378

[67] Besse B Solomon BJ Felip E Bauer TM Ou S-HI Soo RA. Lorlatinib in patients (Pts) with previously treated ALK+ advanced non-small cell lung cancer (NSCLC): Updated efficacy and safety. J Clin Oncol. (2018). doi: 10.1200/JCO.2018.36.15_suppl.9032

[68] Shaw AT Solomon BJ Besse B Bauer TM Lin CC Soo RA. ALK resistance mutations and efficacy of lorlatinib in advanced anaplastic lymphoma kinase-positive non-small-cell lung cancer. J Clin Oncol. (2019) 37:1370–9. doi: 10.1200/JCO.18.02236 PMC6544460 30892989

[69] Shaw AT Bauer TM de Marinis F Felip E Goto Y Liu G. First-line lorlatinib or crizotinib in advanced ALK-positive lung cancer. N Engl J Med. (2020) 383:2018–29. doi: 10.1056/NEJMoa2027187 33207094

[70] Solomon BJ Bauer TM Ignatius Ou SH Liu G Hayashi H Bearz A. *Post hoc* analysis of lorlatinib intracranial efficacy and safety in patients with ALK-positive advanced non-small-cell lung cancer from the phase III CROWN study. J Clin Oncol. (2022) 40:3593–602. doi: 10.1200/JCO.21.02278 PMC9622589 35605188

[71] Baik CS Myall NJ Wakelee HA. Targeting BRAF-mutant non-small cell lung cancer: from molecular profiling to rationally designed therapy. Oncologist. (2017) 22:786–96. doi: 10.1634/theoncologist.2016-0458 PMC5507646 28487464

[72] Nguyen-Ngoc T Bouchaab H Adjei AA Peters S. BRAF alterations as therapeutic targets in non-small-cell lung cancer. J Thorac Oncol. (2015) 10:1396–403. doi: 10.1097/JTO.0000000000000644 26301799

[73] Herbst RS Morgensztern D Boshoff C. The biology and management of non-small cell lung cancer. Nature. (2018) 553:446–54. doi: 10.1038/nature25183 29364287

[74] Litvak AM Paik PK Woo KM Sima CS Hellmann MD Arcila ME. Clinical characteristics and course of 63 patients with BRAF mutant lung cancers. J Thorac Oncol. (2014) 9:1669–74. doi: 10.1097/JTO.0000000000000344 PMC4251710 25436800

[75] Marchetti A Felicioni L Malatesta S Grazia Sciarrotta M Guetti L Chella A. Clinical features and outcome of patients with non-small-cell lung cancer harboring BRAF mutations. J Clin Oncol. (2011) 29:3574–9. doi: 10.1200/JCO.2011.35.9638 21825258

[76] Planchard D Smit EF Groen HJM Mazieres J Besse B Helland Å. Dabrafenib plus trametinib in patients with previously untreated BRAF(V600E)-mutant metastatic non-small-cell lung cancer: an open-label, phase 2 trial. Lancet Oncol. (2017) 18:1307–16. doi: 10.1016/S1470-2045(17)30679-4 28919011

[77] O’Leary CG Andelkovic V Ladwa R Pavlakis N Zhou C Hirsch F. Targeting BRAF mutations in non-small cell lung cancer. Transl Lung Cancer Res. (2019) 8:1119–24. doi: 10.21037/tlcr.2019.10.22 PMC6976351 32010589

[78] Riely GJ Smit EF Ahn MJ Felip E Ramalingam SS Tsao A. Open-label study of encorafenib plus binimetinib in patients with BRAF(V600)-mutant metastatic non-small-cell lung cancer. J Clin Oncol. (2023) 41:3700–11. doi: 10.1200/JCO.23.00774 37270692

[79] Li BT Shen R Buonocore D Olah ZT Ni A Ginsberg MS. Ado-trastuzumab emtansine for patients with HER2-mutant lung cancers: results from a phase II basket trial. J Clin Oncol. (2018) 36:2532–7. doi: 10.1200/JCO.2018.77.9777 PMC6366814 29989854

[80] Li BT Shen R Buonocore D Olah ZT Ni A Ginsberg MS. Ado-trastuzumab emtansine in patients with HER2 mutant lung cancers: Results from a phase II basket trial. J Clin Oncol. (2017). doi: 10.1200/JCO.2017.35.15_suppl.8510 PMC6366814 29989854

[81] Iwama E Zenke Y Sugawara S Daga H Morise M Yanagitani N. Trastuzumab emtansine for patients with non-small cell lung cancer positive for human epidermal growth factor receptor 2 exon-20 insertion mutations. Eur J Cancer. (2022) 162:99–106. doi: 10.1016/j.ejca.2021.11.021 34959152

[82] Li BT Smit EF Goto Y Nakagawa K Udagawa H Mazières J. Trastuzumab deruxtecan in HER2-mutant non-small-cell lung cancer. N Engl J Med. (2022) 386:241–51. doi: 10.1056/NEJMoa2112431 PMC9066448 34534430

[83] Tsurutani J Iwata H Krop I Jänne PA Doi T Takahashi S. Targeting HER2 with trastuzumab deruxtecan: A dose-expansion, phase I study in multiple advanced solid tumors. Cancer Discovery. (2020) 10:688–701. doi: 10.1158/2159-8290.CD-19-1014 32213540 PMC8292921

[84] Skoulidis F Heymach JV. Co-occurring genomic alterations in non-small-cell lung cancer biology and therapy. Nat Rev Cancer. (2019) 19:495–509. doi: 10.1038/s41568-019-0179-8 31406302 PMC7043073

[85] Dogan S Shen R Ang DC Johnson ML D’Angelo SP Paik PK. Molecular epidemiology of EGFR and KRAS mutations in 3,026 lung adenocarcinomas: higher susceptibility of women to smoking-related KRAS-mutant cancers. Clin Cancer Res. (2012) 18:6169–77. doi: 10.1158/1078-0432.CCR-11-3265 PMC3500422 23014527

[86] Canon J Rex K Saiki AY Mohr C Cooke K Bagal D. The clinical KRAS(G12C) inhibitor AMG 510 drives anti-tumour immunity. Nature. (2019) 575:217–23. doi: 10.1038/s41586-019-1694-1 31666701

[87] Nakajima EC Drezner N Li X Mishra-Kalyani PS Liu Y Zhao H. FDA approval summary: sotorasib for KRAS G12C-mutated metastatic NSCLC. Clin Cancer Res. (2022) 28:1482–6. doi: 10.1158/1078-0432.CCR-21-3074 PMC9012672 34903582

[88] Skoulidis F Li BT Dy GK Price TJ Falchook GS Wolf J. Sotorasib for lung cancers with KRAS p.G12C mutation. N Engl J Med. (2021) 384:2371–81. doi: 10.1056/NEJMoa2103695 PMC9116274 34096690

[89] de Langen AJ Johnson ML Mazieres J Dingemans AC Mountzios G Pless M. Sotorasib versus docetaxel for previously treated non-small-cell lung cancer with KRAS(G12C) mutation: a randomised, open-label, phase 3 trial. Lancet. (2023) 401:733–46. doi: 10.1016/S0140-6736(23)00221-0 36764316

[90] Jänne PA Riely GJ Gadgeel SM Heist RS Ou SI Pacheco JM. Adagrasib in non-small-cell lung cancer harboring a KRAS(G12C) mutation. N Engl J Med. (2022) 387:120–31. doi: 10.1056/NEJMoa2204619 35658005

[91] Wolf J Seto T Han JY Reguart N Garon EB Groen HJM. Capmatinib in MET exon 14-mutated or MET-amplified non-small-cell lung cancer. N Engl J Med. (2020) 383:944–57. doi: 10.1056/NEJMoa2002787 32877583

[92] Garon EB Heist RS Seto T Han J-Y Reguart N Groen HJ. Abstract CT082: Capmatinib in MET ex14-mutated (mut) advanced non-small cell lung cancer (NSCLC): Results from the phase II GEOMETRY mono-1 study, including efficacy in patients (pts) with brain metastases (BM). Cancer Res. (2020) 80:CT082–2. doi: 10.1158/1538-7445.AM2020-CT082

[93] Choi W Park SY Lee Y Lim KY Park M Lee GK. The clinical impact of capmatinib in the treatment of advanced non-small cell lung cancer with MET exon 14 skipping mutation or gene amplification. Cancer Res Treat. (2021) 53:1024–32. doi: 10.4143/crt.2020.1331 PMC8524022 33540494

[94] Drilon A Clark JW Weiss J Ou SI Camidge DR Solomon BJ. Antitumor activity of crizotinib in lung cancers harboring a MET exon 14 alteration. Nat Med. (2020) 26:47–51. doi: 10.1038/s41591-019-0716-8 31932802 PMC8500676

[95] Camidge DR Otterson GA Clark JW Ignatius Ou SH Weiss J Ades S. Crizotinib in patients with MET-amplified NSCLC. J Thorac Oncol. (2021) 16:1017–29. doi: 10.1016/j.jtho.2021.02.010 33676017

[96] Paik PK Felip E Veillon R Sakai H Cortot AB Garassino MC. Tepotinib in non-small-cell lung cancer with MET exon 14 skipping mutations. N Engl J Med. (2020) 383:931–43. doi: 10.1056/NEJMoa2004407 PMC8422679 32469185

[97] Le X Paz-Ares LG Van Meerbeeck J Viteri S Galvez CC Smit EF. Tepotinib in patients with non-small cell lung cancer with high-level MET amplification detected by liquid biopsy: VISION Cohort B. Cell Rep Med. (2023) 4:101280. doi: 10.1016/j.xcrm.2023.101280 37944528 PMC10694660

[98] Drilon A Laetsch TW Kummar S DuBois SG Lassen UN Demetri GD. Efficacy of larotrectinib in TRK fusion-positive cancers in adults and children. N Engl J Med. (2018) 378:731–9. doi: 10.1056/NEJMoa1714448 PMC5857389 29466156

[99] Doebele RC Drilon A Paz-Ares L Siena S Shaw AT Farago AF. Entrectinib in patients with advanced or metastatic NTRK fusion-positive solid tumours: integrated analysis of three phase 1-2 trials. Lancet Oncol. (2020) 21:271–82. doi: 10.1016/S1470-2045(19)30691-6 PMC7461630 31838007

[100] Drilon A Barlesi F Braud FD Cho BC Ahn M-J Siena S. Abstract CT192: Entrectinib in locally advanced or metastatic ROS1 fusion-positive non-small cell lung cancer (NSCLC): Integrated analysis of ALKA-372-001, STARTRK-1 and STARTRK-2. Cancer Res. (2019) 79:CT192–2. doi: 10.1158/1538-7445.AM2019-CT192

[101] Doebele R Ahn M Siena S Drilon A Krebs M Lin C. OA02. 01 Efficacy and safety of entrectinib in locally advanced or metastatic ROS1 fusion-positive non-small cell lung cancer (NSCLC). J Thorac Oncol. (2018) 13:S321–2. doi: 10.1016/j.jtho.2018.08.239 PMC8078299 33646820

[102] Lassen U Albert CM Kummar S Van Tilburg C DuBois SG Geoerger B. Larotrectinib efficacy and safety in TRK fusion cancer: an expanded clinical dataset showing consistency in an age and tumor agnostic approach. Ann Oncol. (2018) 29:viii133. doi: 10.1093/annonc/mdy279.397

[103] Collisson EA Campbell JD Brooks AN Berger AH Lee W Chmielecki J. Comprehensive molecular profiling of lung adenocarcinoma. Nature. (2014) 511:543–50. doi: 10.1038/nature13385 PMC4231481 25079552

[104] Bronte G Ulivi P Verlicchi A Cravero P Delmonte A Crinò L. Targeting RET-rearranged non-small-cell lung cancer: future prospects. Lung Cancer (Auckl). (2019) 10:27–36. doi: 10.2147/LCTT 30962732 PMC6433115

[105] Gainor JF Curigliano G Kim DW Lee DH Besse B Baik CS. Pralsetinib for RET fusion-positive non-small-cell lung cancer (ARROW): a multi-cohort, open-label, phase 1/2 study. Lancet Oncol. (2021) 22:959–69. doi: 10.1016/S1470-2045(21)00247-3 34118197

[106] Wright KM. FDA approves pralsetinib for treatment of adults with metastatic RET fusion-positive NSCLC. Oncol (Williston Park). (2020) 34:406–6. doi: 10.46883/ONCOLOGY 33058106

[107] Drilon A Oxnard GR Tan DSW Loong HHF Johnson M Gainor J. Efficacy of selpercatinib in RET fusion-positive non-small-cell lung cancer. N Engl J Med. (2020) 383:813–24. doi: 10.1056/NEJMoa2005653 PMC7506467 32846060

[108] Drilon A Oxnard G Wirth L Besse B Gautschi O Tan S. PL02. 08 registrational results of LIBRETTO-001: a phase 1/2 trial of LOXO-292 in patients with RET fusion-positive lung cancers. J Thorac Oncol. (2019) 14:S6–7.

[109] Drilon A Rekhtman N Arcila M Wang L Ni A Albano M. Cabozantinib in patients with advanced RET-rearranged non-small-cell lung cancer: an open-label, single-centre, phase 2, single-arm trial. Lancet Oncol. (2016) 17:1653–60. doi: 10.1016/S1470-2045(16)30562-9 PMC5143197 27825636

[110] Drilon A Wang L Hasanovic A Suehara Y Lipson D Stephens P. Response to Cabozantinib in patients with RET fusion-positive lung adenocarcinomas. Cancer Discovery. (2013) 3:630–5. doi: 10.1158/2159-8290.CD-13-0035 PMC4160032 23533264

[111] Lin JJ Shaw AT. Recent advances in targeting ROS1 in lung cancer. J Thorac Oncol. (2017) 12:1611–25. doi: 10.1016/j.jtho.2017.08.002 PMC5659942 28818606

[112] Dziadziuszko R Le AT Wrona A Jassem J Camidge DR Varella-Garcia M. An activating KIT mutation induces crizotinib resistance in ROS1-positive lung cancer. J Thorac Oncol. (2016) 11:1273–81. doi: 10.1016/j.jtho.2016.04.001 PMC4961521 27068398

[113] Wu YL Yang JC Kim DW Lu S Zhou J Seto T. Phase II study of crizotinib in east asian patients with ROS1-positive advanced non-small-cell lung cancer. J Clin Oncol. (2018) 36:1405–11. doi: 10.1200/JCO.2017.75.5587 29596029

[114] Shaw AT Ou SH Bang YJ Camidge DR Solomon BJ Salgia R. Crizotinib in ROS1-rearranged non-small-cell lung cancer. N Engl J Med. (2014) 371:1963–71. doi: 10.1056/NEJMoa1406766 PMC4264527 25264305

[115] Michels S Massutí B Schildhaus H-U Franklin J Sebastian M Felip E. Safety and efficacy of crizotinib in patients with advanced or metastatic ROS1-rearranged lung cancer (EUCROSS): a European phase II clinical trial. J Thorac Oncol. (2019) 14:1266–76. doi: 10.1016/j.jtho.2019.03.020 30978502

[116] Mazières J Zalcman G Crinò L Biondani P Barlesi F Filleron T. Crizotinib therapy for advanced lung adenocarcinoma and a ROS1 rearrangement: results from the EUROS1 cohort. J Clin Oncol. (2015) 33:992–9. doi: 10.1200/JCO.2014.58.3302 25667280

[117] Drilon A Siena S Dziadziuszko R Barlesi F Krebs MG Shaw AT. Entrectinib in ROS1 fusion-positive non-small-cell lung cancer: integrated analysis of three phase 1-2 trials. Lancet Oncol. (2020) 21:261–70. doi: 10.1016/S1470-2045(19)30690-4 PMC7811790 31838015

[118] Drilon A Camidge DR Lin JJ Kim SW Solomon BJ Dziadziuszko R. Repotrectinib in ROS1 fusion-positive non-small-cell lung cancer. N Engl J Med. (2024) 390:118–31. doi: 10.1056/NEJMoa2302299 PMC11702311 38197815

[119] Sandler A Gray R Perry MC Brahmer J Schiller JH Dowlati A. Paclitaxel-carboplatin alone or with bevacizumab for non-small-cell lung cancer. N Engl J Med. (2006) 355:2542–50. doi: 10.1056/NEJMoa061884 17167137

[120] Saito H Fukuhara T Furuya N Watanabe K Sugawara S Iwasawa S. Erlotinib plus bevacizumab versus erlotinib alone in patients with EGFR-positive advanced non-squamous non-small-cell lung cancer (NEJ026): interim analysis of an open-label, randomised, multicentre, phase 3 trial. Lancet Oncol. (2019) 20:625–35. doi: 10.1016/S1470-2045(19)30035-X 30975627

[121] Nakagawa K Garon EB Seto T Nishio M Ponce Aix S Paz-Ares L. Ramucirumab plus erlotinib in patients with untreated, EGFR-mutated, advanced non-small-cell lung cancer (RELAY): a randomised, double-blind, placebo-controlled, phase 3 trial. Lancet Oncol. (2019) 20:1655–69. doi: 10.1016/S1470-2045(19)30634-5 31591063

[122] Garon EB Ciuleanu TE Arrieta O Prabhash K Syrigos KN Goksel T. Ramucirumab plus docetaxel versus placebo plus docetaxel for second-line treatment of stage IV non-small-cell lung cancer after disease progression on platinum-based therapy (REVEL): a multicentre, double-blind, randomised phase 3 trial. Lancet. (2014) 384:665–73. doi: 10.1016/S0140-6736(14)60845-X 24933332

[123] Hilberg F Roth GJ Krssak M Kautschitsch S Sommergruber W Tontsch-Grunt U. BIBF 1120: triple angiokinase inhibitor with sustained receptor blockade and good antitumor efficacy. Cancer Res. (2008) 68:4774–82. doi: 10.1158/0008-5472.CAN-07-6307 18559524

[124] Reck M Kaiser R Mellemgaard A Douillard JY Orlov S Krzakowski M. Docetaxel plus nintedanib versus docetaxel plus placebo in patients with previously treated non-small-cell lung cancer (LUME-Lung 1): a phase 3, double-blind, randomised controlled trial. Lancet Oncol. (2014) 15:143–55. doi: 10.1016/S1470-2045(13)70586-2 24411639

[125] Gainor JF Shaw AT. Emerging paradigms in the development of resistance to tyrosine kinase inhibitors in lung cancer. J Clin Oncol. (2013) 31:3987–96. doi: 10.1200/JCO.2012.45.2029 PMC3805932 24101047

[126] Zhang W Lei P Dong X Xu C. The new concepts on overcoming drug resistance in lung cancer. Drug Des Devel Ther. (2014) 8:735–44. doi: 10.2147/DDDT PMC4057322 24944510

[127] Engelman JA Jänne PA. Mechanisms of acquired resistance to epidermal growth factor receptor tyrosine kinase inhibitors in non-small cell lung cancer. Clin Cancer Res. (2008) 14:2895–9. doi: 10.1158/1078-0432.CCR-07-2248 18483355

[128] Pao W Chmielecki J. Rational, biologically based treatment of EGFR-mutant non-small-cell lung cancer. Nat Rev Cancer. (2010) 10:760–74. doi: 10.1038/nrc2947 PMC3072803 20966921

[129] Ellis LM Hicklin DJ. Resistance to targeted therapies: refining anticancer therapy in the era of molecular oncology. Clin Cancer Res. (2009) 15:7471–8. doi: 10.1158/1078-0432.CCR-09-1070 20008847

[130] Oser MG Niederst MJ Sequist LV Engelman JA. Transformation from non-small-cell lung cancer to small-cell lung cancer: molecular drivers and cells of origin. Lancet Oncol. (2015) 16:e165–72. doi: 10.1016/S1470-2045(14)71180-5 PMC4470698 25846096

[131] Rath B Plangger A Hamilton G. Non-small cell lung cancer-small cell lung cancer transformation as mechanism of resistance to tyrosine kinase inhibitors in lung cancer. Cancer Drug Resist. (2020) 3:171–8. doi: 10.20517/cdr.2019.85 PMC9090586 35582610

[132] Jackman D Pao W Riely GJ Engelman JA Kris MG Jänne PA. Clinical definition of acquired resistance to epidermal growth factor receptor tyrosine kinase inhibitors in non-small-cell lung cancer. J Clin Oncol. (2010) 28:357–60. doi: 10.1200/JCO.2009.24.7049 PMC3870288 19949011

[133] Yasuda H Kobayashi S Costa DB. EGFR exon 20 insertion mutations in non-small-cell lung cancer: preclinical data and clinical implications. Lancet Oncol. (2012) 13:e23–31. doi: 10.1016/S1470-2045(11)70129-2 21764376

[134] Pao W Miller VA Politi KA Riely GJ Somwar R Zakowski MF. Acquired resistance of lung adenocarcinomas to gefitinib or erlotinib is associated with a second mutation in the EGFR kinase domain. PloS Med. (2005) 2:e73. doi: 10.1371/journal.pmed.0020073 15737014 PMC549606

[135] Yu PP Vose JM Hayes DF. Genetic cancer susceptibility testing: increased technology, increased complexity. J Clin Oncol. (2015) 33:3533–4. doi: 10.1200/JCO.2015.63.3628 26324366

[136] Yu HA Arcila ME Rekhtman N Sima CS Zakowski MF Pao W. Analysis of tumor specimens at the time of acquired resistance to EGFR-TKI therapy in 155 patients with EGFR-mutant lung cancers. Clin Cancer Res. (2013) 19:2240–7. doi: 10.1158/1078-0432.CCR-12-2246 PMC3630270 23470965

[137] Riely GJ Yu HA. EGFR: the paradigm of an oncogene-driven lung cancer. Clin Cancer Res. (2015) 21:2221–6. doi: 10.1158/1078-0432.CCR-14-3154 PMC4435716 25979928

[138] Finlay MR Anderton M Ashton S Ballard P Bethel PA Box MR. Discovery of a potent and selective EGFR inhibitor (AZD9291) of both sensitizing and T790M resistance mutations that spares the wild type form of the receptor. J Med Chem. (2014) 57:8249–67. doi: 10.1021/jm500973a 25271963

[139] Kosaka T Yatabe Y Endoh H Yoshida K Hida T Tsuboi M. Analysis of epidermal growth factor receptor gene mutation in patients with non-small cell lung cancer and acquired resistance to gefitinib. Clin Cancer Res. (2006) 12:5764–9. doi: 10.1158/1078-0432.CCR-06-0714 17020982

[140] Onitsuka T Uramoto H Nose N Takenoyama M Hanagiri T Sugio K. Acquired resistance to gefitinib: the contribution of mechanisms other than the T790M, MET, and HGF status. Lung Cancer. (2010) 68:198–203. doi: 10.1016/j.lungcan.2009.05.022 19589612

[141] Yun CH Mengwasser KE Toms AV Woo MS Greulich H Wong KK. The T790M mutation in EGFR kinase causes drug resistance by increasing the affinity for ATP. Proc Natl Acad Sci U.S.A. (2008) 105:2070–5. doi: 10.1073/pnas.0709662105 PMC2538882 18227510

[142] Huang L Fu L. Mechanisms of resistance to EGFR tyrosine kinase inhibitors. Acta Pharm Sin B. (2015) 5:390–401. doi: 10.1016/j.apsb.2015.07.001 26579470 PMC4629442

[143] Bean J Riely GJ Balak M Marks JL Ladanyi M Miller VA. Acquired resistance to epidermal growth factor receptor kinase inhibitors associated with a novel T854A mutation in a patient with EGFR-mutant lung adenocarcinoma. Clin Cancer Res. (2008) 14:7519–25. doi: 10.1158/1078-0432.CCR-08-0151 PMC2596620 19010870

[144] Costa DB Halmos B Kumar A Schumer ST Huberman MS Boggon TJ. BIM mediates EGFR tyrosine kinase inhibitor-induced apoptosis in lung cancers with oncogenic EGFR mutations. PloS Med. (2007) 4:1669–79. doi: 10.1371/journal.pmed.0040315 PMC2043012 17973572

[145] Toyooka S Date H Uchida A Kiura K Takata M. The epidermal growth factor receptor D761Y mutation and effect of tyrosine kinase inhibitor. Clin Cancer Res. (2007) 13:3431. doi: 10.1158/1078-0432.CCR-07-0070 17545553

[146] Leonetti A Sharma S Minari R Perego P Giovannetti E Tiseo M. Resistance mechanisms to osimertinib in EGFR-mutated non-small cell lung cancer. Br J Cancer. (2019) 121:725–37. doi: 10.1038/s41416-019-0573-8 PMC6889286 31564718

[147] Takeda M Nakagawa K. First- and second-generation EGFR-TKIs are all replaced to osimertinib in chemo-naive EGFR mutation-positive non-small cell lung cancer? Int J Mol Sci. (2019) 20. doi: 10.3390/ijms20010146 PMC6337322 30609789

[148] Heuckmann JM Balke-Want H Malchers F Peifer M Sos ML Koker M. Differential protein stability and ALK inhibitor sensitivity of EML4-ALK fusion variants. Clin Cancer Res. (2012) 18:4682–90. doi: 10.1158/1078-0432.CCR-11-3260 22912387

[149] Choi YL Soda M Yamashita Y Ueno T Takashima J Nakajima T. EML4-ALK mutations in lung cancer that confer resistance to ALK inhibitors. N Engl J Med. (2010) 363:1734–9. doi: 10.1056/NEJMoa1007478 20979473

[150] Doebele RC Pilling AB Aisner DL Kutateladze TG Le AT Weickhardt AJ. Mechanisms of resistance to crizotinib in patients with ALK gene rearranged non-small cell lung cancer. Clin Cancer Res. (2012) 18:1472–82. doi: 10.1158/1078-0432.CCR-11-2906 PMC3311875 22235099

[151] Kim S Kim TM Kim DW Go H Keam B Lee SH. Heterogeneity of genetic changes associated with acquired crizotinib resistance in ALK-rearranged lung cancer. J Thorac Oncol. (2013) 8:415–22. doi: 10.1097/JTO.0b013e318283dcc0 23344087

[152] Ai X Niu X Chang L Chen R Ou SI Lu S. Next generation sequencing reveals a novel ALK G1128A mutation resistant to crizotinib in an ALK-Rearranged NSCLC patient. Lung Cancer. (2018) 123:83–6. doi: 10.1016/j.lungcan.2018.07.004 30089600

[153] Yanagitani N Uchibori K Koike S Tsukahara M Kitazono S Yoshizawa T. Drug resistance mechanisms in Japanese anaplastic lymphoma kinase-positive non-small cell lung cancer and the clinical responses based on the resistant mechanisms. Cancer Sci. (2020) 111:932–9. doi: 10.1111/cas.14314 PMC7060465 31961053

[154] Dehghanian F Kay M Vallian S. F1174V mutation alters the ALK active conformation in response to Crizotinib in NSCLC: Insight from molecular simulations. J Mol Graph Model. (2017) 75:287–93. doi: 10.1016/j.jmgm.2017.06.010 28622610

[155] Katayama R Shaw AT Khan TM Mino-Kenudson M Solomon BJ Halmos B. Mechanisms of acquired crizotinib resistance in ALK-rearranged lung Cancers. Sci Transl Med. (2012) 4:120ra17. doi: 10.1126/scitranslmed.3003316 PMC3385512 22277784

[156] Novello S Mazières J Oh IJ de Castro J Migliorino MR Helland Å. Alectinib versus chemotherapy in crizotinib-pretreated anaplastic lymphoma kinase (ALK)-positive non-small-cell lung cancer: results from the phase III ALUR study. Ann Oncol. (2018) 29:1409–16. doi: 10.1093/annonc/mdy121 PMC6005013 29668860

[157] Yang Y Zhou J Zhou J Feng J Zhuang W Chen J. Efficacy, safety, and biomarker analysis of ensartinib in crizotinib-resistant, ALK-positive non-small-cell lung cancer: a multicentre, phase 2 trial. Lancet Respir Med. (2020) 8:45–53. doi: 10.1016/S2213-2600(19)30252-8 31628085

[158] Camidge DR Kim DW Tiseo M Langer CJ Ahn MJ Shaw AT. Exploratory analysis of brigatinib activity in patients with anaplastic lymphoma kinase-positive non-small-cell lung cancer and brain metastases in two clinical trials. J Clin Oncol. (2018) 36:2693–701. doi: 10.1200/JCO.2017.77.5841 29768119

[159] Gainor JF Dardaei L Yoda S Friboulet L Leshchiner I Katayama R. Molecular mechanisms of resistance to first- and second-generation ALK inhibitors in ALK-rearranged lung cancer. Cancer Discovery. (2016) 6:1118–33. doi: 10.1158/2159-8290.CD-16-0596 PMC5050111 27432227

[160] Pailler E Faugeroux V Oulhen M Mezquita L Laporte M Honoré A. Acquired resistance mutations to ALK inhibitors identified by single circulating tumor cell sequencing in ALK-rearranged non-small-cell lung cancer. Clin Cancer Res. (2019) 25:6671–82. doi: 10.1158/1078-0432.CCR-19-1176 31439588

[161] Sasaki T Koivunen J Ogino A Yanagita M Nikiforow S Zheng W. A novel ALK secondary mutation and EGFR signaling cause resistance to ALK kinase inhibitors. Cancer Res. (2011) 71:6051–60. doi: 10.1158/0008-5472.CAN-11-1340 PMC3278914 21791641

[162] Lovly CM McDonald NT Chen H Ortiz-Cuaran S Heukamp LC Yan Y. Rationale for co-targeting IGF-1R and ALK in ALK fusion-positive lung cancer. Nat Med. (2014) 20:1027–34. doi: 10.1038/nm.3667 PMC4159407 25173427

[163] Crystal AS Shaw AT Sequist LV Friboulet L Niederst MJ Lockerman EL. Patient-derived models of acquired resistance can identify effective drug combinations for cancer. Science. (2014) 346:1480–6. doi: 10.1126/science.1254721 PMC4388482 25394791

[164] Tsuji T Ozasa H Aoki W Aburaya S Funazo T Furugaki K. Alectinib resistance in ALK-rearranged lung cancer by dual salvage signaling in a clinically paired resistance model. Mol Cancer Res. (2019) 17:212–24. doi: 10.1158/1541-7786.MCR-18-0325 30171175

[165] Shi R Filho SNM Li M Fares A Weiss J Pham NA. BRAF V600E mutation and MET amplification as resistance pathways of the second-generation anaplastic lymphoma kinase (ALK) inhibitor alectinib in lung cancer. Lung Cancer. (2020) 146:78–85. doi: 10.1016/j.lungcan.2020.05.018 32521388

[166] Dagogo-Jack I Yoda S Lennerz JK Langenbucher A Lin JJ Rooney MM. MET alterations are a recurring and actionable resistance mechanism in ALK-positive lung cancer. Clin Cancer Res. (2020) 26:2535–45. doi: 10.1158/1078-0432.CCR-19-3906 PMC7269872 32086345

[167] Gainor JF Tseng D Yoda S Dagogo-Jack I Friboulet L Lin JJ. Patterns of metastatic spread and mechanisms of resistance to crizotinib in ROS1-positive non-small-cell lung cancer. JCO Precis Oncol. (2017) 2017. doi: 10.1200/PO.17.00063 PMC5766287 29333528

[168] McCoach CE Le AT Gowan K Jones K Schubert L Doak A. Resistance mechanisms to targeted therapies in ROS1(+) and ALK(+) non-small cell lung cancer. Clin Cancer Res. (2018) 24:3334–47. doi: 10.1158/1078-0432.CCR-17-2452 PMC6050099 29636358

[169] D’Angelo A Sobhani N Chapman R Bagby S Bortoletti C Traversini M. Focus on ROS1-positive non-small cell lung cancer (NSCLC): crizotinib, resistance mechanisms and the newer generation of targeted therapies. Cancers (Basel). (2020) 12. doi: 10.3390/cancers12113293 PMC7694780 33172113

[170] Lin JJ Choudhury NJ Yoda S Zhu VW Johnson TW Sakhtemani R. Spectrum of mechanisms of resistance to crizotinib and lorlatinib in ROS1 fusion-positive lung cancer. Clin Cancer Res. (2021) 27:2899–909. doi: 10.1158/1078-0432.CCR-21-0032 PMC8127383 33685866

[171] Zhou Y Jiang W Zeng L Mi J Song L Lizaso A. A novel ROS1 G2032 K missense mutation mediates lorlatinib resistance in a patient with ROS1-rearranged lung adenocarcinoma but responds to nab-paclitaxel plus pembrolizumab. Lung Cancer. (2020) 143:55–9. doi: 10.1016/j.lungcan.2020.03.019 32208297

[172] Facchinetti F Loriot Y Kuo MS Mahjoubi L Lacroix L Planchard D. Crizotinib-resistant ROS1 mutations reveal a predictive kinase inhibitor sensitivity model for ROS1- and ALK-rearranged lung cancers. Clin Cancer Res. (2016) 22:5983–91. doi: 10.1158/1078-0432.CCR-16-0917 27401242

[173] Facchinetti F Rossi G Bria E Soria JC Besse B Minari R. Oncogene addiction in non-small cell lung cancer: Focus on ROS1 inhibition. Cancer Treat Rev. (2017) 55:83–95. doi: 10.1016/j.ctrv.2017.02.010 28342334

[174] Drilon A Somwar R Wagner JP Vellore NA Eide CA Zabriskie MS. A novel crizotinib-resistant solvent-front mutation responsive to cabozantinib therapy in a patient with ROS1-rearranged lung cancer. Clin Cancer Res. (2016) 22:2351–8. doi: 10.1158/1078-0432.CCR-15-2013 PMC4867287 26673800

[175] Zou HY Li Q Engstrom LD West M Appleman V Wong KA. PF-06463922 is a potent and selective next-generation ROS1/ALK inhibitor capable of blocking crizotinib-resistant ROS1 mutations. Proc Natl Acad Sci U.S.A. (2015) 112:3493–8. doi: 10.1073/pnas.1420785112 PMC4371934 25733882

[176] Song A Kim TM Kim DW Kim S Keam B Lee SH. Molecular changes associated with acquired resistance to crizotinib in ROS1-rearranged non-small cell lung cancer. Clin Cancer Res. (2015) 21:2379–87. doi: 10.1158/1078-0432.CCR-14-1350 25688157

[177] Drilon A Jenkins C Iyer S Schoenfeld A Keddy C Davare MA. ROS1-dependent cancers - biology, diagnostics and therapeutics. Nat Rev Clin Oncol. (2021) 18:35–55. doi: 10.1038/s41571-020-0408-9 32760015 PMC8830365

[178] Zhu YC Lin XP Li XF Wu LX Chen HF Wang WX. Concurrent ROS1 gene rearrangement and KRAS mutation in lung adenocarcinoma: A case report and literature review. Thorac Cancer. (2018) 9:159–63. doi: 10.1111/1759-7714.12518 PMC5754306 28971587

[179] Park K Ahn M Yu C Kim S Lin M Sriuranpong V. ASPIRATION: first-line erlotinib (E) until and beyond RECIST progression (PD) in Asian patients (pts) with EGFR mutation-positive (mut+) NSCLC. Ann Oncol. (2014) 25:iv426. doi: 10.1093/annonc/mdu349.2

[180] Ou SH Jänne PA Bartlett CH Tang Y Kim DW Otterson GA. Clinical benefit of continuing ALK inhibition with crizotinib beyond initial disease progression in patients with advanced ALK-positive NSCLC. Ann Oncol. (2014) 25:415–22. doi: 10.1093/annonc/mdt572 24478318

[181] Wu YL Zhang L Kim DW Liu X Lee DH Yang JC. Phase ib/II study of capmatinib (INC280) plus gefitinib after failure of epidermal growth factor receptor (EGFR) inhibitor therapy in patients with EGFR-mutated, MET factor-dysregulated non-small-cell lung cancer. J Clin Oncol. (2018) 36:3101–9. doi: 10.1200/JCO.2018.77.7326 30156984

[182] Yang JJ Fang J Shu YQ Chang JH Chen GY He JX. A phase Ib study of the highly selective MET-TKI savolitinib plus gefitinib in patients with EGFR-mutated, MET-amplified advanced non-small-cell lung cancer. Invest New Drugs. (2021) 39:477–87. doi: 10.1007/s10637-020-01010-4 33052556

[183] Wu YL Cheng Y Zhou J Lu S Zhang Y Zhao J. Tepotinib plus gefitinib in patients with EGFR-mutant non-small-cell lung cancer with MET overexpression or MET amplification and acquired resistance to previous EGFR inhibitor (INSIGHT study): an open-label, phase 1b/2, multicentre, randomised trial. Lancet Respir Med. (2020) 8:1132–43. doi: 10.1016/S2213-2600(20)30154-5 32479794

[184] Sequist LV Han JY Ahn MJ Cho BC Yu H Kim SW. Osimertinib plus savolitinib in patients with EGFR mutation-positive, MET-amplified, non-small-cell lung cancer after progression on EGFR tyrosine kinase inhibitors: interim results from a multicentre, open-label, phase 1b study. Lancet Oncol. (2020) 21:373–86. doi: 10.1016/S1470-2045(19)30785-5 32027846

[185] Hanna NH Schneider BJ Temin S Baker S Jr. Brahmer J Ellis PM. Therapy for stage IV non-small-cell lung cancer without driver alterations: ASCO and OH (CCO) joint guideline update. J Clin Oncol. (2020) 38:1608–32. doi: 10.1200/JCO.19.03022 31990617

[186] Paz-Ares L Luft A Vicente D Tafreshi A Gümüş M Mazières J. Pembrolizumab plus chemotherapy for squamous non-small-cell lung cancer. N Engl J Med. (2018) 379:2040–51. doi: 10.1056/NEJMoa1810865 30280635

[187] Socinski MA Jotte RM Cappuzzo F Orlandi F Stroyakovskiy D Nogami N. Atezolizumab for first-line treatment of metastatic nonsquamous NSCLC. N Engl J Med. (2018) 378:2288–301. doi: 10.1056/NEJMoa1716948 29863955

[188] Gogishvili M Melkadze T Makharadze T Giorgadze D Dvorkin M Penkov K. Cemiplimab plus chemotherapy versus chemotherapy alone in non-small cell lung cancer: a randomized, controlled, double-blind phase 3 trial. Nat Med. (2022) 28:2374–80. doi: 10.1038/s41591-022-01977-y PMC9671806 36008722

[189] Sezer A Kilickap S Gümüş M Bondarenko I Özgüroğlu M Gogishvili M. Cemiplimab monotherapy for first-line treatment of advanced non-small-cell lung cancer with PD-L1 of at least 50%: a multicentre, open-label, global, phase 3, randomised, controlled trial. Lancet. (2021) 397:592–604. doi: 10.1016/s0140-6736(21)00228-2 33581821

[190] Borghaei H Paz-Ares L Horn L Spigel DR Steins M Ready NE. Nivolumab versus docetaxel in advanced nonsquamous non-small-cell lung cancer. N Engl J Med. (2015) 373:1627–39. doi: 10.1056/NEJMoa1507643 PMC5705936 26412456

[191] Brahmer J Reckamp KL Baas P Crinò L Eberhardt WE Poddubskaya E. Nivolumab versus docetaxel in advanced squamous-cell non-small-cell lung cancer. N Engl J Med. (2015) 373:123–35. doi: 10.1056/NEJMoa1504627 PMC4681400 26028407

[192] Gainor JF Shaw AT Sequist LV Fu X Azzoli CG Piotrowska Z. EGFR mutations and ALK rearrangements are associated with low response rates to PD-1 pathway blockade in non-small cell lung cancer: A retrospective analysis. Clin Cancer Res. (2016) 22:4585–93. doi: 10.1158/1078-0432.CCR-15-3101 PMC5026567 27225694

[193] Rittmeyer A Barlesi F Waterkamp D Park K Ciardiello F von Pawel J. Atezolizumab versus docetaxel in patients with previously treated non-small-cell lung cancer (OAK): a phase 3, open-label, multicentre randomised controlled trial. Lancet. (2017) 389:255–65. doi: 10.1016/S0140-6736(16)32517-X PMC6886121 27979383

[194] Herbst RS Baas P Kim DW Felip E Pérez-Gracia JL Han JY. Pembrolizumab versus docetaxel for previously treated, PD-L1-positive, advanced non-small-cell lung cancer (KEYNOTE-010): a randomised controlled trial. Lancet. (2016) 387:1540–50. doi: 10.1016/S0140-6736(15)01281-7 26712084

